# Heart Rate Variability Monitoring During Strength and High-Intensity Interval Training Overload Microcycles

**DOI:** 10.3389/fphys.2019.00582

**Published:** 2019-05-22

**Authors:** Christoph Schneider, Thimo Wiewelhove, Christian Raeder, Andrew A. Flatt, Olaf Hoos, Laura Hottenrott, Oliver Schumbera, Michael Kellmann, Tim Meyer, Mark Pfeiffer, Alexander Ferrauti

**Affiliations:** ^1^Department of Training and Exercise Science, Faculty of Sport Science, Ruhr University Bochum, Bochum, Germany; ^2^Department of Health Sciences and Kinesiology, Biodynamics and Human Performance Center, Georgia Southern University, Savannah, GA, United States; ^3^Center for Sports and Physical Education, Julius-Maximilians-University, Würzburg, Germany; ^4^Hertha Berliner Sport-Club, Berlin, Germany; ^5^Unit of Sport Psychology, Faculty of Sport Science, Ruhr University Bochum, Bochum, Germany; ^6^School of Human Movement and Nutrition Sciences, The University of Queensland, St. Lucia, QLD, Australia; ^7^Institute of Sports and Preventive Medicine, Saarland University, Saarbrücken, Germany; ^8^Department Theory and Practice of Sports, Institute of Sport Science, Johannes Gutenberg University Mainz, Mainz, Germany

**Keywords:** orthostatic test, cardiac autonomic nervous system, fatigue, recovery, individual response, multivariate analysis, resistance training, overreaching

## Abstract

**Objective:** In two independent study arms, we determine the effects of strength training (ST) and high-intensity interval training (HIIT) overload on cardiac autonomic modulation by measuring heart rate (HR) and vagal heart rate variability (HRV).

**Methods:** In the study, 37 well-trained athletes (ST: 7 female, 12 male; HIIT: 9 female, 9 male) were subjected to orthostatic tests (HR and HRV recordings) each day during a 4-day baseline period, a 6-day overload microcycle, and a 4-day recovery period. Discipline-specific performance was assessed before and 1 and 4 days after training.

**Results:** Following ST overload, supine HR, and vagal HRV (Ln RMSSD) were clearly increased and decreased (small effects), respectively, and the standing recordings remained unchanged. In contrast, HIIT overload resulted in decreased HR and increased Ln RMSSD in the standing position (small effects), whereas supine recordings remained unaltered. During the recovery period, these responses were reversed (ST: small effects, HIIT: trivial to small effects). The correlations between changes in HR, vagal HRV measures, and performance were weak or inconsistent. At the group and individual levels, moderate to strong negative correlations were found between HR and Ln RMSSD when analyzing changes between testing days (ST: supine and standing position, HIIT: standing position) and individual time series, respectively. Use of rolling 2–4-day averages enabled more precise estimation of mean changes with smaller confidence intervals compared to single-day values of HR or Ln RMSSD. However, the use of averaged values displayed unclear effects for evaluating associations between HR, vagal HRV measures, and performance changes, and have the potential to be detrimental for classification of individual short-term responses.

**Conclusion:** Measures of HR and Ln RMSSD during an orthostatic test could reveal different autonomic responses following ST or HIIT which may not be discovered by supine or standing measures alone. However, these autonomic changes were not consistently related to short-term changes in performance and the use of rolling averages may alter these relationships differently on group and individual level.

## Introduction

Efficient training provides sufficient exercise stimuli to enhance athletes' performance capacities while avoiding sustained non-functional overreaching or underrecovery (Meeusen et al., 2013; Kellmann et al., 2018). Continuous athlete monitoring may provide information that can be used to balance stress and recovery, ultimately increasing the performance readiness of the athlete and minimizing the risk of illness and injury (Halson, 2014; Schwellnus et al., 2016; Soligard et al., 2016; Bourdon et al., 2017; Coutts et al., 2018; Heidari et al., 2019). A variety of tools, such as psychometric questionnaires (Kellmann, 2010; Saw et al., 2016), blood-borne markers (Urhausen et al., 1995; Fry and Kraemer, 1997; Urhausen and Kindermann, 2002; Meeusen et al., 2013), heart rate (HR)-based measures (Achten and Jeukendrup, 2003; Aubert et al., 2003; Bosquet et al., 2008; Meeusen et al., 2013; Buchheit, 2014; Bellenger et al., 2016a), and (submaximal or non-fatiguing) performance tests (Urhausen and Kindermann, 2002; Meeusen et al., 2013; Claudino et al., 2017), have been discussed for their potential as surrogate markers for assessing fatigue, recovery, or performance. Ideally, so that they can be used frequently in sports practice, these measures are non-fatiguing, easy to administer, inexpensive, and sensitive to performance changes and can provide immediate feedback (Starling and Lambert, 2018).

Monitoring the status of the autonomic nervous system (ANS) with HR-based measures [HR and HR variability (HRV) indices] is an attractive option for testing due to its non-invasiveness and time-efficiency when performed for an entire training group or team. Technological developments within the last few decades have enabled practitioners to use portable devices to obtain accurate beat-by-beat recordings (Achten and Jeukendrup, 2003; Quintana et al., 2012; Buchheit, 2014) as well as software and smart phone applications (Flatt and Esco, 2013; Perrotta et al., 2017; Plews et al., 2017) to obtain (almost) live feedback on HR and HRV indices [HR(V)] in the field. In applied sports research and practice, HR (or average R-R interval) and the time-domain HRV marker RMSSD (root mean square of successive differences between adjacent beat to beat intervals) are commonly measured in a supine, seated, or standing position and may indicate training and fatigue status (Buchheit, 2014; Schmitt et al., 2015a; Bellenger et al., 2016a; Thorpe et al., 2017). However, as ANS activity, and therefore HRV indices, are determined by multiple factors (Sandercock et al., 2005; Buchheit, 2014; Fatisson et al., 2016), it remains difficult to interpret HR(V) measures in isolation. This may contribute to the partially contradictory findings in the literature (Buchheit, 2014; Schneider et al., 2018). To overcome some of these limitations, it has been proposed that researchers use a combination of supine and standing recordings during an orthostatic test to discriminate between different fatigue patterns (Bosquet et al., 2008; Schmitt et al., 2013, 2015a,b; Hottenrott and Hoos, 2017) and use rolling averages to assess adaptation to training (Le Meur et al., 2013; Plews et al., 2014; Flatt and Esco, 2015).

Individuals' HR(V) responses likely differ with training context (i.e., training phase and history, exercise modality and intensity, and the time course of response) (Stanley et al., 2013; Buchheit, 2014; Schmitt et al., 2015a). The majority of relevant scientific reviews focus on either endurance-trained athletes (Bosquet et al., 2008; Bellenger et al., 2016a) or non-athletic populations (Kingsley and Figueroa, 2016; Bhati et al., 2018). Based on this background and to extend insights into training context-specific HR(V) responses, we conducted two independent, similarly designed training trials using whole-body strength training (ST) or high-intensity interval training (HIIT) overload microcycles in well-trained athletes. This study aims to (1) determine the mean changes in HR and HRV measures during active orthostatic tests following ST and HIIT overload and subsequent short-term recovery; (2) evaluate the association between changes in HR, HRV, and performance; (3) classify individuals' HR(V) responses; and (4) analyze whether the use of single-day vs. two-day to four-day average HR values affects the results of the three main analyses.

## Materials and Methods

### Participants

Initially, 55 athletes were recruited, of which 51 met the inclusion criteria and 45 competed the study (5 participants did not meet compliance criteria, one dropout due to injury). From the original samples of 23 (9 female, 14 male) individuals performing ST and 22 (11 female, 11 male) individuals performing HIIT, only 19 (7 female, 12 male) and 18 (9 female, 9 male), respectively, participants provided a sufficient quantity of resting HR recordings to be included in this investigation (we set a minimum of three baseline recordings and a maximum of one missing recording during recovery). The general subject characteristics are presented in Table 1. Preliminary health examinations, including resting and exercise electrocardiograms, confirmed the absence of cardiovascular, pulmonary, or orthopedic diseases.

**Table 1:** Subject characteristics.

	**Age**	**Height**	**Weight**	**BMI**	**V°O_**2**_ peak**	**PTRS**	**Training volume**	
	**(years)**	**(cm)**	**(kg)**	**(kg/m^**2**^)**	**(ml/kg/min)**	**(km/h)**	**(n/week)**	**(h/week)**
**STRENGTH TRAINING**								
female (*n =* 7)	25.0 ± 1.5	167.0 ± 4.9	62.0 ± 7.0	22.2 ± 1.4	45.5 ± 4.5	14.5 ± 1.3	4.1 ± 1.3	7.9 ± 3.5
male (*n =* 12)	24.1 ± 2.2	179.9 ± 5.4	77.8 ± 6.7	24.0 ± 1.7	56.6 ± 4.8	16.6 ± 0.8	4.2 ± 1.5	7.2 ± 3.1
overall (*n =* 19)	24.4 ± 2.0	175.2 ± 8.2	71.9 ± 10.2	23.3 ± 1.8	52.5 ± 7.1	15.8 ± 1.5	4.2 ± 1.4	7.4 ± 3.2
**HIGH-INTENSITY INTERVAL TRAINING**								
female (*n =* 9)	27.1 ± 3.6	171.3 ± 3.8	64.0 ± 5.1	21.8 ± 1.4	53.1 ± 5.0	15.1 ± 1.6	4.0 ± 1.8	5.3 ± 3.6
male (*n =* 9)	27.0 ± 2.1	180.8 ± 5.4	73.7 ± 6.7	22.6 ± 2.7	63.5 ± 8.8	17.7 ± 2.0	4.3 ± 1.7	6.7 ± 3.5
overall (*n =* 18)	27.1 ± 2.8	176.1 ± 6.7	68.8 ± 7.6	22.2 ± 2.1	58.3 ± 8.8	16.4 ± 2.2	4.2 ± 1.7	6.0 ± 3.5

*Data are shown as mean ± standard deviation. BMI, body mass index; $\dot{V}$ O_2_peak, peak oxygen uptake; PTRS, peak treadmill running speed*.

The following inclusion criteria were used for the ST group: estimated one-repetition maximum (1RM) for a parallel squat of at least 80% of body mass for females and 120% of body mass for males and a minimum of 3 years of lower-body strength training with at least two strength training sessions per week. The inclusion criteria for the HIIT group were as follows: peak velocity during the 30-15 Intermittent Fitness Test (V_IFT_) of at least 16 km/h for females and 19 km/h for males, and a minimum of 5 years of team sport training.

The investigation was approved by the ethics committee of the medical faculty of the Ruhr University Bochum and was conducted according to the guidelines of the Declaration of Helsinki. All subjects participated in the study voluntarily, were free to withdraw without penalty at any time, and provided written informed consent. Participation was rewarded with 100 € at the end of the investigation.

### Experimental Design

**Figure 1:**
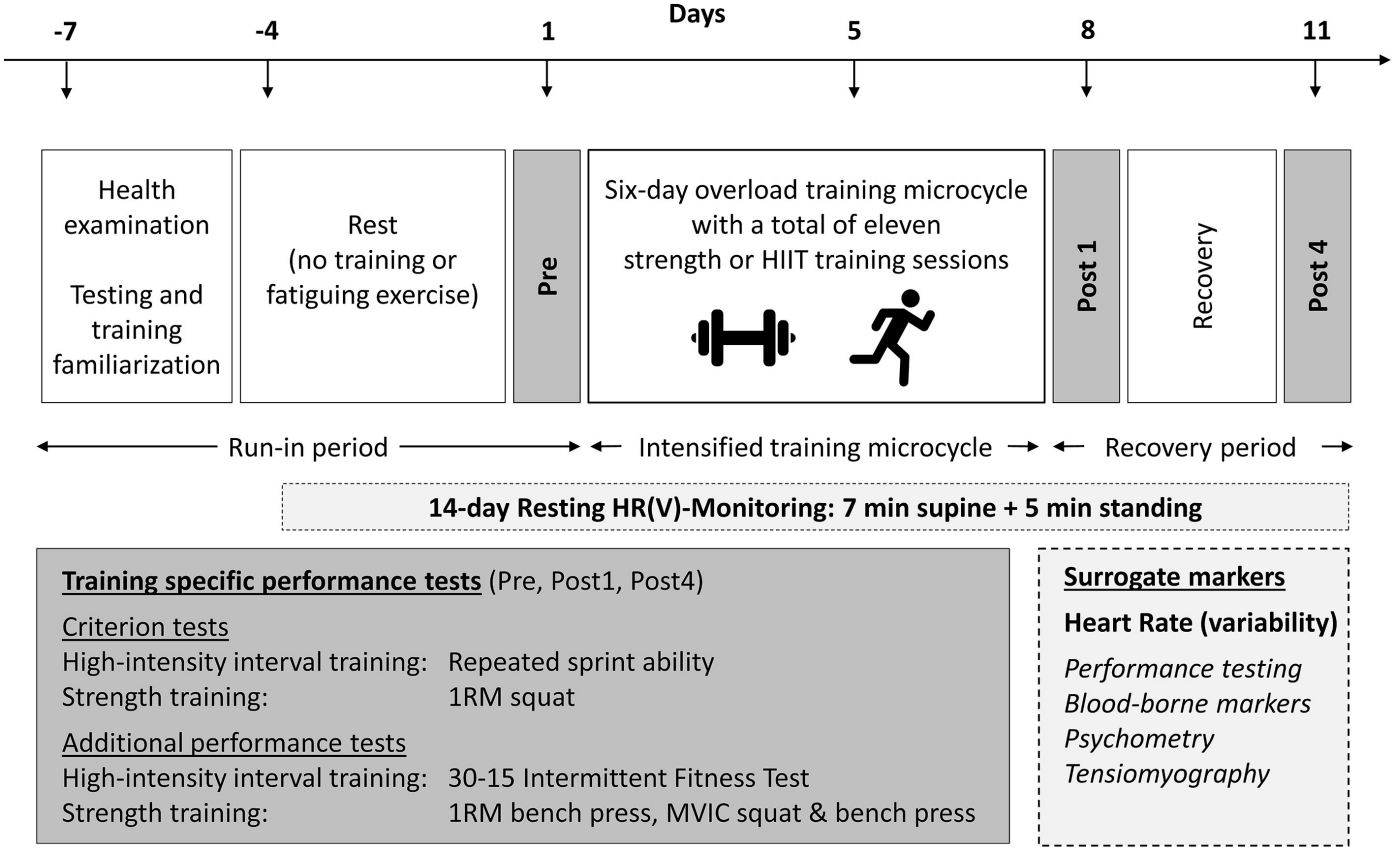
Experimental design. HIIT, high-intensity interval training; HR(V), heart rate (variability); MVIC, maximum voluntary isometric contraction; 1RM, one-repetition maximum. Surrogate measures in italics are measures that were published previously and are not part of this article (see Methods section).

A repeated-measures study was used to investigate the effects of short-term fatigue and recovery on resting HR and HRV measures [HR(V)]. The investigation comprised a 3-day rest period, baseline testing (Pre), a 6-day overload microcycle, and a 4-day recovery period, which included follow-up testing at 1 (Post1) and 4 (Post4) days post-training (Figure 1). Overload was induced by either intensive whole-body ST or HIIT in two independent study arms. Health examination (incl. survey of medication and nutritional supplementation), determination of peak oxygen consumption ($\dot{V}$O_2_peak)and familiarization trials for training and testing procedures were conducted 1 week before baseline testing. Discipline-specific maximum effort tests were used as criterion measures to assess fatigue- and recovery-related changes in performance. HR(V) measures were recorded daily during the main 14-day study period (including the rest period, overload training, and recovery). An overview of the experimental design is shown in Figure 1.

The analyses presented below were part of an extensive investigation protocol evaluating the ability of different potential surrogate markers to assess fatigue- and recovery-related changes in criterion performance at various overload training camps (i.e., cycling-based endurance training, running-based HIIT, whole-body ST) using a consistent design. The results regarding the performance tests (Wiewelhove et al., 2015; Hammes et al., 2016; Raeder et al., 2016), blood-borne markers (Hecksteden et al., 2016), psychological measures (Hitzschke et al., 2017), and muscle mechanical properties (de Paula Simola et al., 2016) have already been published. Due to insufficient HR(V) baseline recordings, the cycling-based endurance training study arm could not be considered for analyses. Data is provided as Supplementary Material.

### Training Program

The training microcycles were designed to induce functional overreaching and decrease discipline-specific criterion performance 1 day after training, with the effects reversed on the fourth day post-overload. Two training sessions were performed per day, and on the fourth day of training, the morning session was substituted by an intermediate test of surrogate measures, resulting in a total of 11 training sessions. Table 2 presents a general overview of the training schedules, and more details can be found in other reports (Wiewelhove et al., 2015; Raeder et al., 2016).

**Table 2:** Overload training microcycles.

	**Day 1**	**Day 2**	**Day 3**	**Day 4**	**Day 5**	**Day 6**
**STRENGTH TRAINING**						
a.m.	FW squats	FW squats	FW squats	Intermediate	FW squats	FW squats
	TD bench press	DS bench press	DS bench press	testing	DS bench press	DS bench press
	Hamstrings, back & core	Hamstrings, back & core	Hamstrings, back & core		Hamstrings, back & core	Hamstrings, back & core
p.m.	EO squats	TD squats	EO squats	TD squats	EO squats	TD squats
	TD squats	FW squats	TD squats	FW squats	TD squats	FW squats
	EO bench press	TD bench press	EO bench press	TD bench press	EO bench press	TD bench press
	Hamstrings, back & core	Hamstrings, back & core	Hamstrings, back & core	Hamstrings, back & core	Hamstrings, back & core	Hamstrings, back & core
**Protocol**	**Volume (sets × repetitions)**		**Intensity (% 1RM)**		**Rest**	
TD	4 × 6		85		3 min	
DS	1 × 6 (+3 drop sets)		85 (70-55-40)		30 s	
EO	4 × 6		100 ECC-70 CON		3 min	
FW	4 × 6 (+2 acc reps)		Maximum effort		3 min	
**HIGH-INTENSITY INTERVAL TRAINING**						
a.m.	Straight-line runs	Straight-line runs	Straight-line runs	Intermediate	Straight-line runs	Straight-line runs
	4 × 4 min, 80% V_IFT_	7 × 2 min, 85% V_IFT_	4 × 4 min, 80% V_IFT_	testing	4 × 4 min, 80% V_IFT_	7 × 2 min, 85% V_IFT_
	(*r =* 3 min)	(*r =* 2 min)	(*r =* 3 min)		(*r =* 3 min)	(*r =* 2 min)
p.m.	Straight-line sprints	40m-shuttle runs	Straight-line sprints	40m-shuttle runs	Straight-line sprints	40m-shuttle runs
	4 × 6 × 5 s, all out	2 × 12 × 30 s, 90% V_IFT_	4 × 6 × 5 s, all out	2 × 12 × 30 s, 90% V_IFT_	4 × 6 × 5 s, all out	2 × 12 × 30 s, 90% V_IFT_
	(*r =* 25 s; *R =* 5 min)	(*r =* 30 s; *R =* 3 min)	(*r =* 25 s; *R =* 5 min)	(*r =* 30 s; *R =* 3 min)	(*r =* 25 s; *R =* 5 min)	(*r =* 30 s; *R =* 3 min)

FW, flywheel YoYo squat; TD, traditional multiple sets; DS, drop sets; EO, eccentric overload; 1RM, one-repetition maximum; ECC, eccentric; CON, concentric; acc reps, acceleration repetitions; VIFT, peak running speed obtained in the 30-15 Intermittent Fitness Test; r, passive recovery between intervals; R, passive recovery between series. Raeder et al. (2016), Wiewelhove et al. (2015) doi: 10.1371/journal.pone.0139801.t002

ST combined multi-joint high-resistance training and maximal eccentric strength training, focusing mainly on lower-body exercises (i.e., parallel squats). The training sessions lasted approximately 90 min. They started with a standardized dynamic warm-up, followed by lower-body exercises, and ended with a combination of upper-body, core, hamstring, and back exercises. Preceding the main training exercises, participants performed specific warm-up sets of 5 to 3 repetitions at 50% and 70% of individual maximum performance, respectively. Exercise intensity was standardized in relation to the estimated maximal dynamic strength (1RM) or maximum effort (Table 2).

HIIT included straight-line runs, straight-line sprints, and shuttle runs performed on an outdoor 400 m Tartan track. Training sessions lasted approximately 35 min. They started with a standardized 10-min continuous warm-up consisting of 40-m shuttle runs at approximately 60–70% of participants' maximum HR, followed by four 40-m acceleration sprints. Exercise intensity was standardized in relation to peak velocity during the 30–15 Intermittent Fitness Test (V_IFT_) or maximum effort (Table 2).

### Procedures

#### Performance Measures

A detailed description of the testing procedures can be found in the original publications regarding ST (Raeder et al., 2016) and HIIT (Wiewelhove et al., 2015). On the testing days Pre, Post1, and Post4, participants in the ST overload group were subjected to maximum dynamic and isometric strength tests. Criterion performance was measured by participants' 1RM for parallel squats. On the main testing days, participants in the HIIT overload group were subjected to maximum intermittent shuttle-run test and a repeated sprint ability (RSA) test. RSA was defined as the criterion performance measure.

##### Incremental treadmill test

An incremental treadmill test (Ergo ELG2, Woodway GmbH, Weil am Rhein, Germany) using a breath-by-breath gas collection system (ZAN600USB, nSpire Health GmbH, Oberthulba, Germany) was employed to measure $\dot{V}$O_2_peak in order to characterize the participants aerobic capacity. Initial velocity was set at 8 km/h, with 2 km/h increments introduced every 3 min and a constant incline of 0.5% until voluntary exhaustion. The highest 30 s mean value was defined as the $\dot{V}$O_2_peak.

##### Maximum voluntary isometric contraction

Participants' maximum voluntary isometric contraction (MVIC) force output for parallel squat and bench press exercises was determined using a Multitrainer 7812-000 testing device similar to a Smith machine (Kettler Profiline, Ense-Parsit, Germany) and the corresponding user software (DigiMax, version 7.X). Joint angles were set at 90° using a goniometer and the corresponding testing device position was kept constant throughout the study. Following two submaximal practice trials at ~50 and 70% of participants' MVIC, the participants were asked to produce a 3-s MVIC with initial slow force development. MVIC test performance was defined as the mean force of two attempts separated by a rest of 2 min.

##### One-repetition maximum

Participants' maximum dynamic strength was assessed 60 min after MVIC testing for parallel squat (ST criterion performance measure) and bench press exercises using a Smith rack machine (Technogym, Cesena, Italy). Squat depth was standardized using an integrated linear transducer that produced acoustic stimuli to mark the turning point of the motion. Participants completed two warm-up sets of 5 and 3 repetitions at 50 and 70% of their individual 5–10 RM, respectively. Using a formula by Brzycki (Maud and Foster, 2006), participants' 1RM was estimated based on the heaviest 5–10 RM lift within a maximum of three testing sets separated by rests of 3 min. Tests were stopped when the subjects were unable to raise the barbell using a proper technique or when the supervisors' help was required. The reliability of the 1RM squat test was previously investigated in our laboratory and was determined to be high [1RM (kg), *n* = 38, ICC = 0.96, TE 5.2 (Raeder et al., 2016)]. Participants' 1RM performance was later used to calculate the exercise intensity of the training protocols.

##### Repeated sprint ability

Participants' RSA (HIIT criterion performance measure) was determined using a non-motorized treadmill (Force 3.0, Woodway GmbH, Weil am Rhein, Germany). The participants completed a standardized warm-up prior to the trial. The test consisted of six 4-s maximal sprints beginning from a standing position with passive recovery of 20 s between sprints. The highest velocities measured for each sprint were recorded, and the mean peak velocity was calculated. The reliability of the RSA test was previously investigated in our laboratory and was determined to be high [mean peak velocity (m/s), *n* = 17, ICC = 0.92, TE 0.1 (Wiewelhove et al., 2015)].

##### Intermittent aerobic performance

Maximum intermittent aerobic performance was assessed based on participants' peak running speed (V_IFT_) during the 30-15 Intermittent Fitness Test (Buchheit, 2008). The test was conducted on an outdoor Tartan track. Participants were tasked with running back and forth between two lines set 40 m apart. The shuttle runs were 30 s with 15 s of passive recovery between each run. The initial running speed was set at 8 km/h, with stepped increases of 0.5 km/h every 45 s. Running speed was declared using audio signals, and V_IFT_ was defined as the velocity of the last completed stage. V_IFT_ was later used to calculate the exercise intensity of the training protocols.

#### Heart Rate and Heart Rate Variability

Every morning, an active orthostatic test (7 min supine, 5 min standing) was performed after participants' awoke and emptied their bladder throughout the main 14-day study period. During recordings, the participants were asked to leave their eyes open, breathe calmly, and avoid movement. A general briefing and written guidelines for the orthostatic test were provided before the beginning of the study (see Supplementary Material for details). R-R series were recorded using Polar RS800cx heart rate monitors (Polar Electro, Kempele, Finland), and the data were transferred to the software Polar Pro Trainer 5 (version 5.40.170, Polar Electro, Kempele, Finland). Polar files (.hrm) were then exported and used for further processing. HR, the natural logarithm of RMSSD (Ln RMSSD) and the Ln RMSSD to R-R interval ratio (Ln RMSSD/RR = Ln RMSSD divided by the mean R-R interval) were calculated during the last 5 min for which participants were supine and the 5-min standing measurements using Kubios (version 2.2, Biosignal Analysis and Medical Imaging Group, University of Eastern Finland, Finland) (Tarvainen et al., 2014). The Ln RMSSD, which is considered to be measure of vagal-mediated HRV (at least in the acute-term) (Malik et al., 1996; Carter et al., 2003), was chosen as the primary HRV marker because it features high reliability (Al Haddad et al., 2011) and is less affected by different breathing patterns compared to spectral analysis (Penttilä et al., 2001; Saboul et al., 2013). Additionally, average HR was calculated (Plews et al., 2013; Buchheit, 2014) to further allow comparison of whether HRV is more sensitive to overload- and recovery-related changes than HR. It remains unclear whether HRV is more sensitive than HR to changes in athletes' training status (Billman et al., 2015b; Schneider et al., 2018). Finally, as previously proposed, the Ln RMSSD/RR was determined to gain further insights into the association between HRV and average HR (Plews et al., 2013; Buchheit, 2014; Billman et al., 2015b; Trimmel et al., 2015, Editorial).

### Data Analysis and Statistical Analysis

Data are presented as mean ± standard deviation (SD) unless otherwise specified. For our statistical analyses, we used Microsoft Excel 2016 (Microsoft Office 365, Version 1810, Microsoft Corp., Redmond, WA, USA) for basic calculations and descriptive statistics and the free open-source software JASP (Version 0.8.6, Amsterdam, Netherlands) (JASP Team, 2018) for inferential procedures. The Shapiro-Wilks test was used to verify the assumed normal distribution of data. Statistical analyses are provided as JASP files (.jasp) in the Supplementary Material.

The day-to-day reliability of resting HR(V) indices were assessed using specifically designed spreadsheets (Hopkins, 2015b). The typical error (TE) was selected as the reliability statistic of interest and was calculated by dividing the standard deviation (SD) of day-to-day differences (SD) by $\sqrt{2}$ pooled for the four-day baseline period (i.e., the differences between days 1 and 2, 2 and 3, and 3 and 4) (Hopkins et al., 2001). The group-based smallest worthwhile change (SWC) was defined as 0.2 × between-subject SD for pooled baseline measurements (Hopkins et al., 2009). TE and SWC were calculated as absolute and percentage values. The spreadsheets used for the assessment are provided as Supplementary Material.

Differences between the three main time points (Pre, Post1, and Post4) were tested by repeated measures analysis of variance (ANOVA; repeated factor: time; grouping factor: sex) including sex as grouping factor to determine possible sex differences in HR(V) responses (Aubert et al., 2003; Sandercock et al., 2005). The violation of sphericity was adjusted by Greenhouse-Geisser correction. Bonferroni-adjusted *p*-values (*p*_bonf_) are reported for pairwise comparisons. The level of significance was set at *p* ≤ 0.05. Further, paired *t*-tests were used to calculate 90% confidence limits (CL) for the mean differences and standardized effect sizes (*d*). The magnitude of change (*d*_pre_) was evaluated using the between-subject Pre-test SD. Then, *d*_pre_ and 90% CL were calculated in MS Excel and adjusted for the sample size using the following formula (Cumming, 2012, p. 294):

(1) $$\begin{array}{r} d\text{ unbiased}=\left(1-\frac{3}{4\;df-1}\right)\times d \end{array}$$

where *df* is the degree of freedom of the SD estimate (*df* = n−1). The threshold values for *d*_pre_ were >0.2 (small), >0.6 (moderate), and >1.2 (large) (Hopkins et al., 2009).

To evaluate the consistency of within-subject changes (*d*_diff_) between the different measures used within our study, mean differences were standardized according to the SD of differences (Dankel and Loenneke, 2018). Then, *d*_diff_ and 90% CL were calculated with JASP.

The Pearson correlation (*r*) and 90% CL were used to evaluate the associations between changes in HR(V) and performance measures as well as between HR and Ln RMSSD. In accordance with previous analyses (Plews et al., 2014), percentage changes from the previous values were correlated for changes between Pre, Post1, and Post4 with JASP. Individual HR and Ln RMSSD time series were correlated with Microsoft Excel. Threshold values for *r* were >0.1 (small), >0.3 (moderate), and >0.5 (large) (Hopkins et al., 2009).

The mean differences and correlations were calculated for changes in daily HR(V) measures and the 2-, 3-, and 4-day rolling averages. When the 90% confidence intervals overlapped small positive and negative values, the effects were deemed *unclear* (Hopkins et al., 2009).

Individual responses were classified as *likely* to be increased or decreased when changes exceeded the TE. Therefore, this category (i.e., “likely”) includes changes for which the approximate 50% confidence interval associated with the observed change (i.e., ±TE) does not include zero change (Hopkins, 2004; Swinton et al., 2018). If changes in ±TE occurred, individual responses were classified as *unclear*. Group-based TE (Wiewelhove et al., 2015; Raeder et al., 2016) was used to assess performance changes, and individual TE (i.e., 4-day baseline SD) was used to classify changes in HR(V) indices. Subsequently, 3 × 3 tables were created to descriptively evaluate the categorial agreement between changes in performance (i.e., criterion) and HR(V) measures (i.e., surrogate) as well as the agreement between changes in HR and Ln RMSSD when using single-day values or 4-day averages, respectively. The evaluation of categorical agreement (i.e., 3 × 3 tables) refers to the commonly proposed threshold-based approaches to assess individual HR(V) changes, for example using the TE or SWC as cut-off values for decision-making (Plews et al., 2013; Buchheit, 2014), and is aimed to complement the assessment of continuous association (i.e., correlations) between HR(V) and performance measures.

## Results

Statistically significant sex differences were apparent (main effect of sex: *p* ≤ 0.05) in the performance data (ST, HIIT) as well as the standing HR (ST, HIIT) and Ln RMSSD (ST) values. In absence of clear interactions between time and sex, statistical analyses were conducted only for pooled data.

### Baseline Recordings and Missing Data

During the baseline period, one supine and/or standing HR(V) recording was missing for three ST and four HIIT participants. Another HR(V) recording for the day following Post1 was missing for one ST participant. This missing data slightly affected the 3- and 4-day rolling averages, but not the degrees of freedom for the analyses.

Descriptively, the day-to-day reliability of HR and Ln RMSSD, expressed as the typical error (TE in %), was larger when the participants were standing compared to supine in the ST group (HR: +2.1%; Ln RMSSD: +1.3%), HIIT group (HR: +2.3%; Ln RMSSD: +5.1%), and pooled data of the ST and HIIT groups (HR: +2.0%; Ln RMSSD: +3.2%) (Table 3). Baseline HR (i.e., 4-day average) was slightly lower for the HIIT group compared to the ST group in a supine position [difference: −3 bpm, smallest worthwhile change (SWC): 1–2 bpm]. However, it was slightly higher when participants were in a standing position (+2 bpm). Differences in Ln RMSSD remained below the SWC (Tables 3–**5**).

**Table 3:** Day-to-day reliability and smallest worthwhile change for resting heart rate (variability) measures during the 4-day baseline period.

		**Typical error**						**Smallest worthwhile change**					
**SUPINE RECORDINGS**													
HR	ST	3	(3; 4)	bpm	5.7	(4.8; 7.1)	%	1	(1; 2)	bpm	2.5	(2.0; 3.5)	%
	HIIT	3	(2; 4)	bpm	4.9	(4.2; 6.3)	%	2	(1; 2)	bpm	2.6	(2.0; 3.6)	%
	pooled	3	(3; 4)	bpm	5.4	(4.8; 6.3)	%	2	(1; 2)	bpm	2.6	(2.1; 3.2)	%
Ln RMSSD	ST	0.24	(0.20; 0.29)	ms	6.0	(5.1; 7.5)	%	0.11	(0.08; 0.15)	ms	2.7	(2.1; 3.7)	%
	HIIT	0.21	(0.17; 0.26)	ms	6.1	(5.1; 7.7)	%	0.11	(0.09; 0.16)	ms	3.1	(2.4; 4.3)	%
	pooled	0.22	(0.20; 0.26)	ms	6.0	(5.3; 7.0)	%	0.11	(0.09; 0.13)	ms	2.8	(2.4; 3.5)	%
Ln RMSSD/RR	ST	0.21	(0.18; 0.26)	×103	5.2	(4.4; 6.4)	%	0.09	(0.07; 0.12)	×103	2.1	(1.7; 2.9)	%
	HIIT	0.28	(0.23; 0.35)	×103	7.4	(6.2; 9.4)	%	0.09	(0.07; 0.12)	×103	2.3	(1.8; 3.2)	%
	pooled	0.26	(0.23; 0.30)	×103	6.7	(5.9; 7.8)	%	0.09	(0.07; 0.11)	×103	2.2	(1.9; 2.8)	%
**STANDING RECORDINGS**													
HR	ST	6	(5; 8)	bpm	7.8	(6.6; 9.7)	%	2	(2; 3)	bpm	2.6	(2.1; 3.7)	%
	HIIT	6	(5; 8)	bpm	7.2	(6.1; 9.2)	%	3	(2; 4)	bpm	3.5	(2.7; 4.9)	%
	pooled	6	(5; 7)	bpm	7.5	(6.6; 8.7)	%	2	(2; 3)	bpm	3.0	(2.5; 3.8)	%
Ln RMSSD	ST	0.24	(0.21; 0.30)	ms	7.2	(6.1; 9.0)	%	0.12	(0.09; 0.16)	ms	3.6	(2.9; 5.1)	%
	HIIT	0.27	(0.23; 0.34)	ms	11.1	(9.4; 14.2)	%	0.14	(0.11; 0.20)	ms	5.6	(4.4; 8.0)	%
	pooled	0.25	(0.22; 0.29)	ms	9.1	(8.1; 10.6)	%	0.13	(0.11; 0.16)	ms	4.7	(3.9; 5.9)	%
Ln RMSSD/RR	ST	0.28	(0.24; 0.35)	×103	6.6	(5.6; 8.2)	%	0.12	(0.09; 0.16)	×103	2.7	(2.1; 3.7)	%
	HIIT	0.22	(0.19; 0.28)	×103	6.5	(5.5; 8.2)	%	0.13	(0.11; 0.19)	×103	3.6	(2.8; 5.1)	%
	pooled	0.25	(0.23; 0.29)	×103	6.5	(5.7; 7.5)	%	0.13	(0.11; 0.16)	×103	3.2	(2.7; 4.0)	%

*Data are shown as mean values and 90% confidence interval. Reliability statistics were calculated for baseline measurements using specifically designed spreadsheet (Hopkins, 2015b). HR, heart rate; Ln RMSSD, natural logarithm of root mean square of successive differences between adjacent beat to beat intervals; Ln RMSSD/RR, Ln RMSSD to R-R interval ratio; ST, strength training (n = 19); HIIT, high-intensity interval training (n = 18), pooled (n = 37). Typical error: Standard deviation of day-to-day differences divided by $\sqrt{2}$ pooled for the 4-day baseline period. Smallest worthwhile change: 0.2 × pooled between-subject SD for baseline measurements*.

### Performance

Participants' 1RM performance in the squat exercise (i.e., criterion) was slightly decreased at Post1 by −4.4 kg (90% CL, −7.8; −1.0 kg) and was increased at Post4 by +4.0 kg (1.0; 7.1 kg). A statistically non-significant main effect was revealed for time (*p* = 0.097). Maximum dynamic and isometric bench press performance showed statistically significant but marginal changes over time (main time effects: *p* ≤ 0.005, |*d*_pre_| ≤ 0.10; Table 4). Repeated sprint ability and maximum intermittent aerobic performance showed small changes over time (main time effects: *p* ≤ 0.005, |*d*_pre_| ≥ 0.27). The mean peak velocity in the RSA test (i.e., criterion) was decreased at Post1 by −0.18 m/s (−0.23; −0.12 m/s) and was increased at Post4 by +0.18 m/s (0.08; 0.27 m/s; Table 5).

**Table 4:** Performance and heart rate (variability) measures at Pre, Post1, Post4, and changes between testing days for the strength training microcycle (7 female, 12 male).

	**Pre**	**Post1**	**Post4**	**Δ Pre to Post1**				**Δ Post1 to Post4**				**Time**
	**Mean ± SD**	**Mean ± SD**	**Mean ± SD**	**Δ ± SD**	***d*_**pre**_ (90% CL)**	***d*_**diff**_**	***p*_**bonf**_**	**Δ ± SD**	***d*_**pre**_ (90% CL)**	***d*_**diff**_**	***p*_**bonf**_**	***p***
**1RM squat (kg)**												
Female	69.1 ± 11.6	66.9 ± 12.0	71.5 ± 15.6									
Male	117.2 ± 23.3	111.4 ± 23.8	115.1 ± 20.3									
Overall	99.5 ± 30.8	95.0 ± 29.7	99.1 ± 28.3	−4.4 ± 8.6	−0.14 (−0.24; −0.03)	−0.52	0.109	4.0 ± 7.6	0.13 (0.03; 0.22)	0.53	0.097	0.097
**MVIC squat (N)**												
Female	952 ± 120	912 ± 81	945 ± 124									
Male	1632 ± 413	1681 ± 370	1705 ± 419									
Overall	1381 ± 472	1398 ± 481	1425 ± 505	17 ± 187	0.03 (−0.12; 0.18)	0.09	1.000	27 ± 191	0.06 (−0.10; 0.21)	0.14	1.000	0.599
**1RM bench (kg)**												
Female	41.9 ± 5.6	41.9 ± 6.7	43.3 ± 7.5									
Male	96.2 ± 16.1	94.2 ± 17.8	97.4 ± 16.7									
OVERALL	76.2 ± 29.9	75.0 ± 29.7	77.4 ± 30.1	−1.2 ± 3.2	−0.04 (−0.08; 0.00)	−0.38	0.356	2.5 ± 3.2	0.08 (0.04; 0.12)	0.78	0.010	0.038
**MVIC bench (N)**												
Female	562 ± 52	539 ± 59	556 ± 62									
Male	1210 ± 197	1164 ± 217	1191 ± 234									
Overall	971 ± 357	933 ± 355	957 ± 366	−38 ± 54	−0.10 (−0.16; −0.04)	−0.70	0.021	24 ± 37	0.06 (0.02; 0.10)	0.63	0.039	0.031
**SUPINE RECORDING**												
**HR (bpm)**												
Single-day	60 ± 8	64 ± 9	61 ± 7	3 ± 7	0.40 (0.06; 0.74)	0.46	0.176	−3 ± 7	−0.34 (−0.65; −0.02)	−0.43	0.236	0.169
2-day avg	61 ± 8	64 ± 9	61 ± 7	3 ± 6	0.36 (0.08; 0.64)	0.50	0.125	−3 ± 5	−0.40 (−0.63; −0.17)	−0.69	0.024	0.048
3-day avg	61 ± 7	64 ± 8	61 ± 7	3 ± 5	0.44 (0.19; 0.68)	0.70	0.021	−3 ± 3	−0.40 (−0.58; −0.22)	−0.87	0.004	0.006
4-day avg	60 ± 7	64 ± 8	62 ± 7	3 ± 4	0.50 (0.28; 0.73)	0.88	0.004	−2 ± 2	−0.30 (−0.39; −0.20)	−1.28	< 0.001	0.005
**Ln RMSSD (ms)**												
Single-day	4.32 ± 0.45	4.08 ± 0.61	4.20 ± 0.46	−0.24 ± 0.42	−0.51 (−0.87; −0.16)	−0.57	0.069	0.12 ± 0.45	0.26 (−0.12; 0.64)	0.27	0.758	0.074
2-day avg	4.28 ± 0.48	4.06 ± 0.57	4.23 ± 0.47	−0.22 ± 0.30	−0.43 (−0.67; −0.20)	−0.73	0.016	0.16 ± 0.30	0.32 (0.08; 0.56)	0.54	0.092	0.018
3-day avg	4.30 ± 0.48	4.08 ± 0.54	4.22 ± 0.45	−0.22 ± 0.23	−0.44 (−0.62; −0.26)	−0.98	0.001	0.14 ± 0.24	0.28 (0.09; 0.47)	0.58	0.063	0.001
4-day avg	4.31 ± 0.46	4.09 ± 0.51	4.18 ± 0.47	−0.22 ± 0.18	−0.45 (−0.60; −0.30)	−1.19	< 0.001	0.09 ± 0.11	0.19 (0.10; 0.28)	0.86	0.004	< 0.001
**STANDING RECORDING**												
**HR (bpm)**												
Single-day	83 ± 10	83 ± 10	85 ± 11	−1 ± 11	−0.07 (−0.51; 0.37)	−0.06	1.000	3 ± 11	0.26 (−0.16; 0.67)	0.25	0.887	0.200
2-day avg	83 ± 9	82 ± 8	83 ± 9	0 ± 6	−0.04 (−0.28; 0.20)	−0.07	1.000	1 ± 7	0.13 (−0.17; 0.44)	0.18	1.000	0.150
3-day avg	82 ± 9	82 ± 8	83 ± 8	0 ± 5	0.01 (−0.21; 0.22)	0.01	1.000	0 ± 5	0.03 (−0.19; 0.25)	0.05	1.000	0.672
4-day avg	82 ± 9	82 ± 8	83 ± 8	1 ± 5	0.07 (−0.14; 0.29)	0.14	1.000	0 ± 4	0.03 (−0.13; 0.19)	0.07	1.000	0.584
**Ln RMSSD (ms)**												
Single-day	3.41 ± 0.46	3.31 ± 0.64	3.22 ± 0.52	−0.09 ± 0.43	−0.19 (−0.54; 0.17)	−0.21	1.000	−0.10 ± 0.45	−0.21 (−0.57; 0.16)	−0.22	1.000	0.160
2-day avg	3.37 ± 0.49	3.35 ± 0.51	3.27 ± 0.52	−0.03 ± 0.19	−0.05 (−0.20; 0.10)	−0.13	1.000	−0.07 ± 0.29	−0.14 (−0.37; 0.08)	−0.26	0.837	0.234
3-day avg	3.37 ± 0.52	3.35 ± 0.55	3.31 ± 0.52	−0.02 ± 0.18	−0.05 (−0.17; 0.08)	−0.14	1.000	−0.04 ± 0.21	−0.06 (−0.22; 0.09)	−0.16	1.000	0.529
4-day avg	3.41 ± 0.52	3.35 ± 0.54	3.31 ± 0.53	−0.06 ± 0.17	−0.11 (−0.24; 0.02)	−0.34	0.460	−0.03 ± 0.15	−0.06 (−0.17; 0.05)	−0.21	1.000	0.190

*Heart rate (variability) measures are provided as single-day values and 2-day to 4-day rolling averages. Data are shown as mean ± SD and 90% confidence limits. 1RM, estimated one-repetition maximum; MVIC, maximum voluntary isometric contraction; HR, heart rate; Ln RMSSD, natural logarithm of root mean square of successive differences between adjacent beat to beat intervals; d_pre_, standardized mean difference using sample-size adjusted, between-subject SD at Pre; d_diff_, standardized mean difference using SD of differences; p_bonf_, Bonferroni adjusted p-values*.

**Table 5:** Performance and heart rate (variability) measures at Pre, Post1, Post4, and changes between testing days for the high-intensity interval training microcycle (9 female, 9 male).

	**Pre**	**Post1**	**Post4**	**Δ Pre to Post1**				**Δ Post1 to Post4**				**Time**
	**Mean ± SD**	**Mean ± SD**	**Mean ± SD**	**Δ ± SD**	***d*_**pre**_ (90% CL)**	***d*_**diff**_**	***p*_**bonf**_**	**Δ ± SD**	***d*_**pre**_ (90% CL)**	***d*_**diff**_**	***p*_**bonf**_**	***p***
**RSA (m/s)**												
Female	4.53 ± 0.19	4.32 ± 0.20	4.57 ± 0.38									
Male	5.49 ± 0.31	5.34 ± 0.28	5.44 ± 0.27									
Overall	5.01 ± 0.55	4.83 ± 0.58	5.01 ± 0.55	−0.18 ± 0.13	−0.31 (−0.40; −0.22)	−1.37	< 0.001	0.18 ± 0.23	0.30 (0.14; 0.47)	0.75	0.016	0.005
**V**_**IFT**_ **(km/h)**												
Female	17.4 ± 1.8	16.8 ± 1.3	17.3 ± 2.1									
Male	20.2 ± 1.3	19.3 ± 1.4	20.0 ± 1.4									
Overall	18.8 ± 2.1	18.1 ± 1.9	18.6 ± 2.2	−0.8 ± 0.7	−0.35 (−0.49; −0.22)	−1.06	< 0.001	0.6 ± 0.8	0.27 (0.12; 0.41)	0.76	0.016	0.001
**SUPINE RECORDING**												
**HR (bpm)**												
Single-day	59 ± 8	57 ± 9	55 ± 8	−1 ± 4	−0.15 (−0.34; 0.05)	−0.30	0.667	−2 ± 4	−0.24 (−0.45; −0.03)	−0.47	0.182	0.004
2-day avg	58 ± 9	59 ± 8	55 ± 8	0 ± 3	0.05 (−0.09; 0.18)	0.14	1.000	−3 ± 3	−0.38 (−0.50; −0.26)	−1.28	< 0.001	< 0.001
3-day avg	58 ± 8	59 ± 9	56 ± 8	1 ± 3	0.16 (0.01; 0.31)	0.43	0.252	−3 ± 2	−0.40 (−0.50; −0.31)	−1.73	< 0.001	< 0.001
4-day avg	57 ± 7	59 ± 8	56 ± 8	1 ± 3	0.19 (0.05; 0.34)	0.56	0.090	−3 ± 2	−0.39 (−0.48; −0.29)	−1.69	< 0.001	< 0.001
**Ln RMSSD (ms)**												
Single day	4.34 ± 0.59	4.38 ± 0.46	4.32 ± 0.51	0.04 ± 0.34	0.06 (−0.17; 0.29)	0.11	1.000	−0.06 ± 0.31	−0.10 (−0.30; 0.11)	−0.19	1.000	0.733
2-day avg	4.26 ± 0.62	4.30 ± 0.50	4.38 ± 0.48	0.04 ± 0.23	0.06 (−0.08; 0.21)	0.18	1.000	0.07 ± 0.27	0.12 (−0.05; 0.28)	0.28	0.765	0.167
3-day avg	4.30 ± 0.56	4.28 ± 0.52	4.37 ± 0.48	−0.02 ± 0.18	−0.04 (−0.17; 0.09)	−0.13	1.000	0.09 ± 0.23	0.15 (−0.01; 0.31)	0.39	0.339	0.182
4-day avg	4.31 ± 0.52	4.26 ± 0.53	4.37 ± 0.46	−0.05 ± 0.18	−0.09 (−0.22; 0.04)	−0.27	0.784	0.11 ± 0.21	0.20 (0.04; 0.36)	0.51	0.138	0.074
**STANDING RECORDING**												
**HR (bpm)**												
Single-day	84 ± 13	76 ± 11	80 ± 13	−8 ± 11	−0.56 (−0.88; −0.25)	−0.74	0.018	4 ± 10	0.31 (0.02; 0.61)	0.44	0.242	0.004
2-day avg	84 ± 13	76 ± 10	80 ± 13	−8 ± 8	−0.59 (−0.82; −0.36)	−1.06	< 0.001	4 ± 9	0.27 (0.01; 0.52)	0.43	0.253	< 0.001
3-day avg	84 ± 13	76 ± 10	79 ± 12	−8 ± 9	−0.56 (−0.81; −0.31)	−0.91	0.004	2 ± 7	0.17 (−0.02; 0.36)	0.36	0.423	< 0.001
4-day avg	84 ± 13	77 ± 9	78 ± 11	−7 ± 9	−0.50 (−0.76; −0.24)	−0.80	0.011	1 ± 6	0.09 (−0.08; 0.26)	0.22	1.000	0.002
**Ln RMSSD (ms)**												
Single-day	3.20 ± 0.59	3.48 ± 0.57	3.36 ± 0.53	0.29 ± 0.36	0.47 (0.23; 0.70)	0.80	0.011	−0.12 ± 0.36	−0.20 (−0.44; 0.04)	−0.34	0.485	0.007
2-day avg	3.16 ± 0.63	3.44 ± 0.51	3.34 ± 0.53	0.27 ± 0.35	0.41 (0.20; 0.63)	0.78	0.012	−0.09 ± 0.39	−0.14 (−0.39; 0.10)	−0.24	0.964	0.015
3-day avg	3.15 ± 0.64	3.44 ± 0.49	3.36 ± 0.52	0.28 ± 0.38	0.42 (0.18; 0.65)	0.73	0.020	−0.07 ± 0.29	−0.11 (−0.28; 0.07)	−0.26	0.879	0.008
4-day avg	3.18 ± 0.64	3.43 ± 0.49	3.39 ± 0.52	0.25 ± 0.37	0.38 (0.15; 0.60)	0.69	0.029	−0.04 ± 0.24	−0.06 (−0.20; 0.09)	−0.16	1.000	0.009

*Heart rate (variability) measures are provided as single-day values and 2-day to 4-day rolling averages. Data are shown as mean ± SD and 90% confidence limits. RSA, mean peak velocity of the repeated sprint ability test; V_IFT_, peak running speed of the 30–15 Intermittent Fitness Test; HR, heart rate; Ln RMSSD, natural logarithm of root mean square of successive differences between adjacent beat to beat intervals; d_pre_, standardized mean difference using sample-size adjusted, between-subject SD at Pre; d_diff_, standardized mean difference using SD of differences; p_bonf_, Bonferroni adjusted p-values*.

### Time Course of HR(V) Measures

For the ST group, daily resting HR increased above the SWC from second day of training to Post1 when participants were in a supine position (effect magnitude range for single day and 2- to 4-day averaged values, ΔPre to Post1: *d*_pre_ = 0.36 to 0.50) and decreased at Post4 (ΔPost1 to Post4: *d*_pre_ = −0.30 to −0.40). The mean supine Ln RMSSD showed an inverse response (ΔPre to Post1: *d*_pre_ = −0.43 to −0.51; ΔPost1 to Post4: *d*_pre_ = 0.19 to 0.32; Table 4). For the HIIT group, daily HR was decreased beyond the SWC from the fourth day of training in the standing position (ΔPre to Post1: *d*_pre_ = −0.50 to −0.59) and increased after Post1 (ΔPost1 to Post4: *d*_pre_ = 0.09 to 0.31). The mean standing Ln RMSSD showed an inverse response (ΔPre to Post1: *d*_pre_ = 0.38 to 0.47; ΔPost1 to Post4: *d*_pre_ = −0.06 to −0.20; Table 5). Changes in Ln RMSSD/RR were unclear for both groups and for both recording positions (see Supplementary Tables 1, 2 for detailed results).

**Figure 2:**
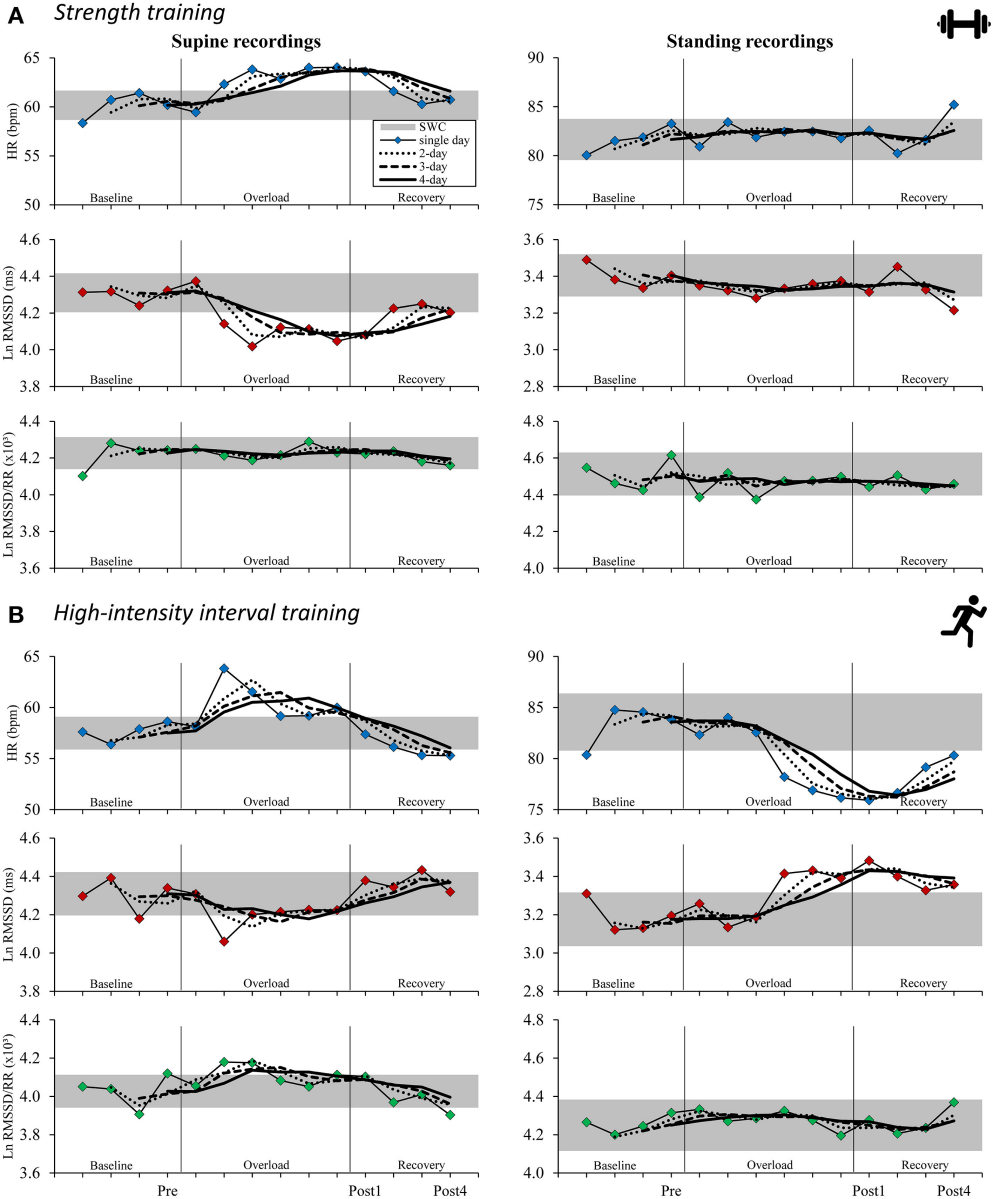
Time course for supine (left) and standing (right) mean resting heart rate (variability) measures in the strength training **(A)** and the high-intensity interval training **(B)** study arms for isolated daily values and rolling 2–4-day averages. Gray horizontal bar: smallest worthwhile change (0.2 × pooled between-subject SD for baseline measurements; see Table 3 for details); single-day: thin lines with colored markings; 2-day rolling average: dotted lines; 3-day rolling average: dashed lines; 4-day rolling average: bold lines. HR, heart rate; Ln RMSSD, natural logarithm of root mean square of successive differences between adjacent beat to beat intervals; Ln RMSSD/RR, Ln RMSSD to R-R interval ratio.

**Figure 3:**
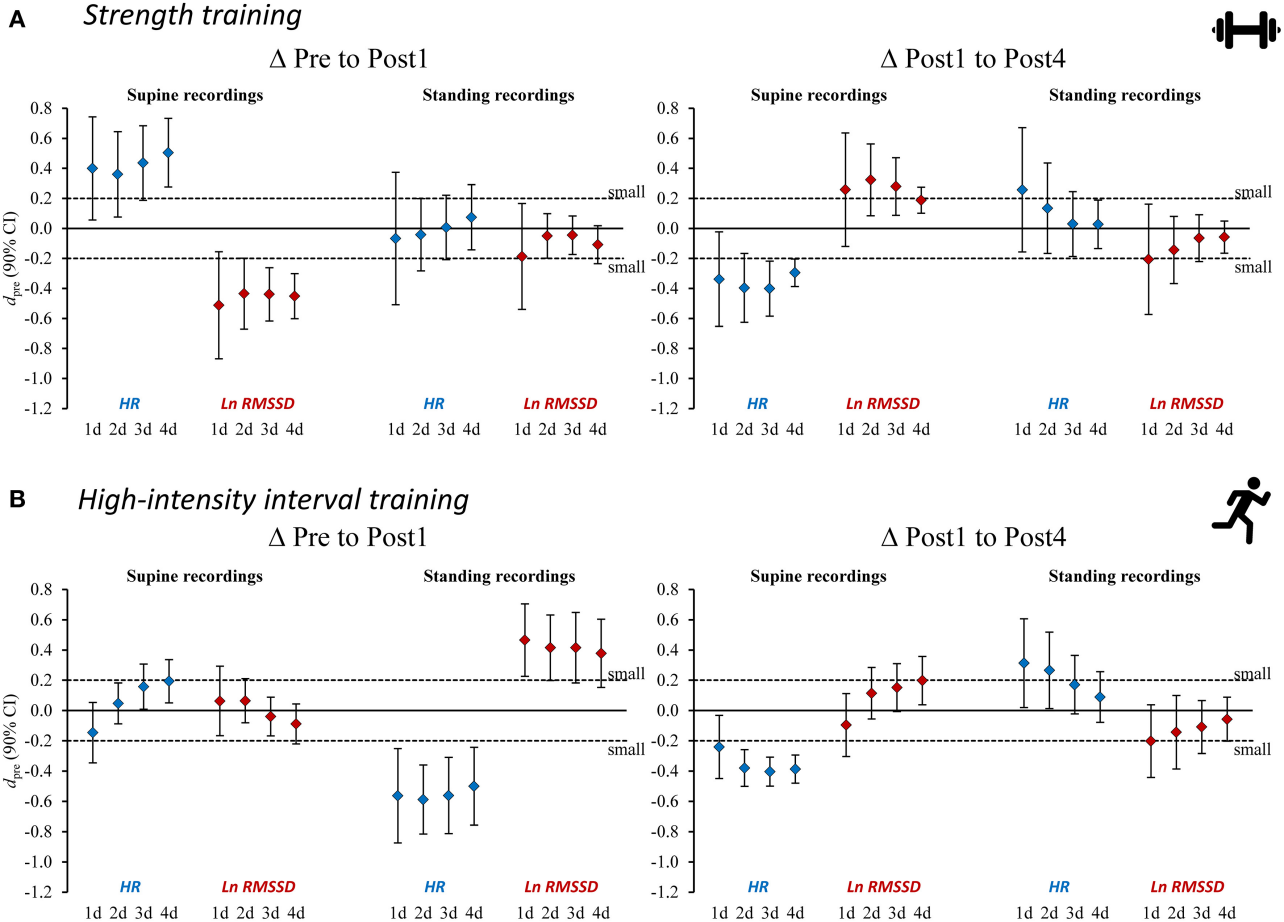
Standardized mean differences (*d*_pre_) for changes in heart rate (variability) measures from Pre to Post1 and from Post 1 to Post4 for **(A)** strength training and **(B)** high-intensity interval training. Heart rate (variability) measures are provided as single-day values and 2–4-day rolling averages. HR, heart rate; Ln RMSSD, natural logarithm of root mean square of successive differences between adjacent beat to beat intervals.

An overview of the time course of mean HR(V) measures is presented in Figure 2 and the mean changes are presented in Tables 4–5 (see Supplementary Tables 1–2 for extensive results). Averaged HR(V) values yielded more precise interval estimates for mean changes (i.e., smaller confidence intervals and larger *d*_diff_) compared to single-day values (Figure 3, Tables 4–5, Supplementary Figure 1, Supplementary Tables 1, 2).

### Association Between Changes in Performance and HR(V)

For both the ST and HIIT groups, the majority of correlation analyses between performance and HR(V) changes were unclear, or inconsistent for changes from Pre to Post1 compared to changes from Post1 to Post4. Moreover, the effect of using average HR(V) values compared to single-day values remained unclear for estimate precision (confidence intervals) and the magnitude (*r*) of correlations. The detailed results of the analyses are presented in Supplementary Figures 2, 3 and Supplementary Tables 3, 4.

### Individual HR(V) Responses

Individual HR(V) responses are presented as single-day and 4-day average values and in reference to the TE as a threshold for response classification for the recording position that was sensitive to group changes (ST: supine recordings; HIIT: standing recordings).

Within the ST group (supine recordings, *n* = 19), Pre to Post1 changes in single-day HR were likely (i.e., beyond ± individual TE) increased in 10 athletes and likely decreased in 3 athletes. Ln RMSSD was likely decreased in 9 athletes and increased in 1 athlete. Of the 10 athletes whose squat 1RM (criterion performance) decreased below group-based TE (4.9%), 5 had likely increased HR and 5 had likely decreased Ln RMSSD. Regarding Pre to Post1 changes in 4-day HR(V) averages, 10 athletes showed likely increased HR and 1 athlete showed likely decreased HR. Ln RMSSD was likely decreased in 8 athletes. Of the 10 athletes whose squat 1RM decreased, 6 had likely increased HR and 4 had likely decreased Ln RMSSD. When classifying individual responses in three categories (likely increased, unclear, likely decreased), changes in squat 1RM and HR(V) agreed with the direction of group changes in the HR of 8 and 9 athletes (single-day and 4-day average, respectively) and in the Ln RMSSD of 9 and 7 athletes (single-day and 4-day average, respectively). In Table 6, blue and gray values represent agreement.

**Table 6:**
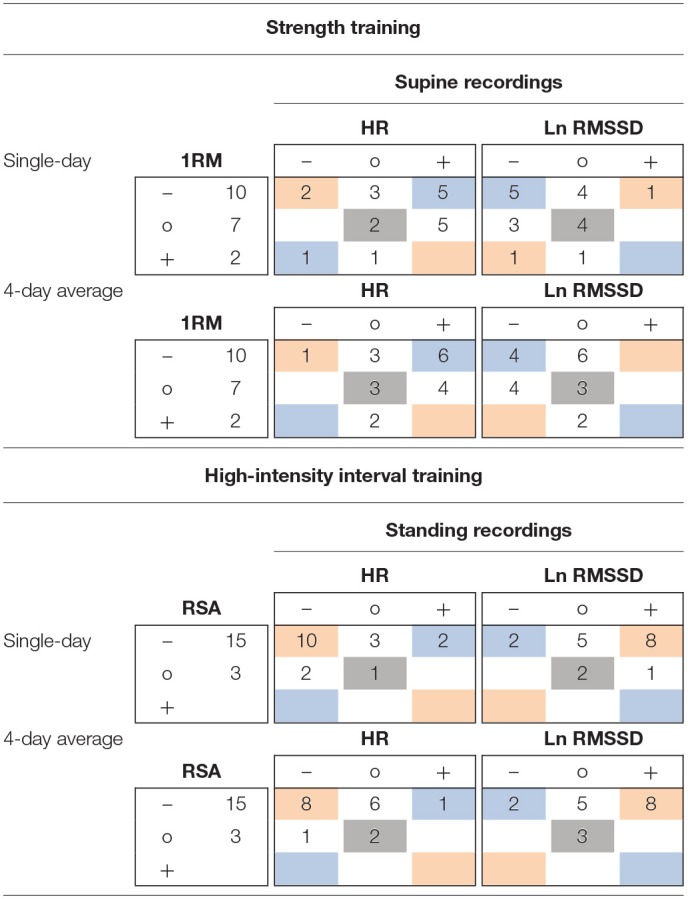
Example 3 × 3 tables for individual response classification for changes from Pre to Post1 of criterion performance and resting heart rate (variability) measures using single-day and 4-day average values.

*Data in the 3 × 3 tables represent the number of observed individual responses classified for 9 response types (3 response types for criterion and heart rate (variability) measures, respectively). HR, heart rate; Ln RMSSD, natural logarithm of root mean square of successive differences between adjacent beat to beat intervals; 1RM, one-repetition maximum in the squat; RSA, repeated sprint ability; –, observed reduction > typical error (TE); o, observed changes < TE; +, observed increase > TE. Group-based TE were used for criterion measures (see Wiewelhove et al., 2015; Raeder et al., 2016) and individual TE was used for heart rate measures (individual SD during 4-day baseline period)*.

**Figure 4:**
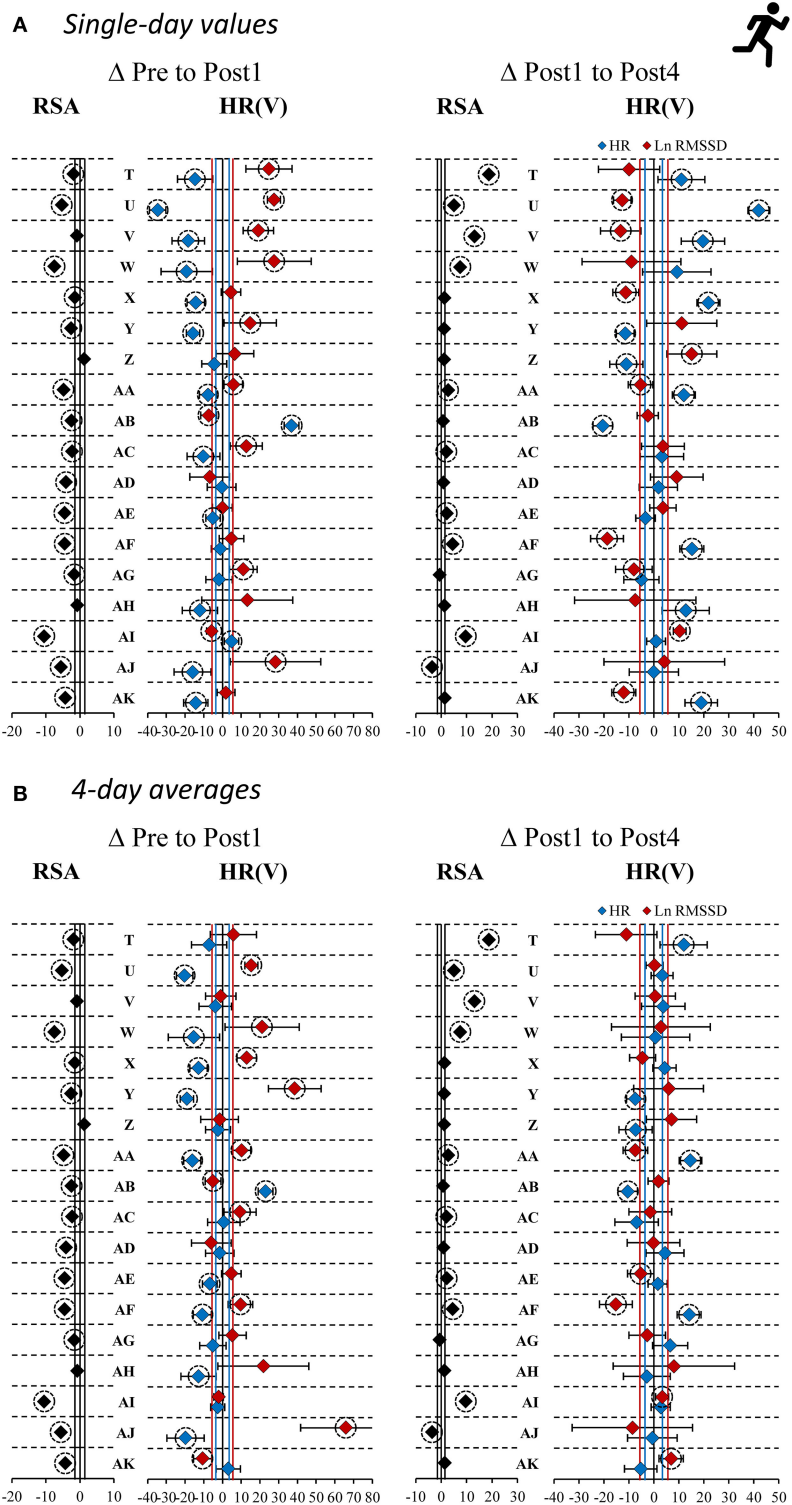
Individual responses as percentage changes in criterion performance and standing heart rate (variability) measures [HR(V)] for high-intensity interval training overload. HR(V) measures are provided as as **(A)** single-day values and **(B)** 4-day rolling averages. RSA, repeated sprint ability; HR, heart rate; Ln RMSSD, natural logarithm of root mean square of successive differences between adjacent beat to beat intervals. Vertical lines: group-based typical error (TE) for RSA (black), smallest worthwhile change (see Table 3) in HR (blue), and Ln RMSSD (red). Error bars: individual TE (4-day baseline SD). Dashed circles: changes exceed ±TE.

For the HIIT group (standing recordings, *n* = 18), Pre to Post1 changes in single-day HR was likely decreased in 12 athletes and likely increased in 2 athletes. Ln RMSSD was likely increased in 9 athletes and decreased in 2 athletes. Of the 15 athletes whose RSA (criterion performance) decreased below group-based TE (1.8%), 10 had likely decreased HR and 8 had likely increased Ln RMSSD. Regarding Pre to Post1 changes in 4-day average HR(V), 9 athletes showed likely decreased HR and 1 athlete showed likely increased HR. Ln RMSSD was likely increased in 8 athletes and likely decreased in 2 athletes. Of the 15 athletes whose RSA decreased, 8 had likely decreased HR and 8 had likely increased Ln RMSSD. When classifying individual responses in three categories (likely increased, unclear, likely decreased), changes in RSA and HR(V) agreed with the direction of group changes in the HR of 11 and 10 athletes (single-day and 4-day average, respectively) and in the Ln RMSSD of 10 and 11 athletes (single-day and 4-day average, respectively). In Table 6, red and gray values represent agreement. Individual responses within the HIIT group (standing recordings) are detailed in Figure 4.

In the ST group (supine recordings), categorial associations between changes in HR and Ln RMSSD showed agreement (i.e., likely increased HR and likely decreased Ln RMSSD, and vice versa) for 11 of 19 athletes (ΔPre to Post1, ΔPost1 to Post4) according to single-day measures. The correlations between HR and Ln RMSSD were moderate to large for changes between main testing days (*r* = −0.33 to −0.77) and large for individual time series (mean *r* = −0.61 to −0.67). In the HIIT group (standing recordings), categorial associations between changes in single-day HR and Ln RMSSD showed agreement for 13 of 18 athletes (ΔPre to Post1) and 12 of 18 athletes (ΔPost1 to Post4). Correlations between HR and Ln RMSSD were trivial to moderate for changes in supine recordings (*r* = −0.35 to 0.06), large for changes in standing recordings (*r* = −0.58 to −0.76), and large for individual time series (mean *r* = −0.50 to −0.78). Most of the time, use of 4-day averages resulted in higher overall categorial agreement and mostly increased agreement for *unclear* changes (i.e., HR and Ln RMSSD changes within ±TE). In Table 7, blue and gray values indicate agreement. A full account of individual responses is provided in the Supplementary Figures 4, 5 and Supplementary Tables 5–7.

**Table 7:**
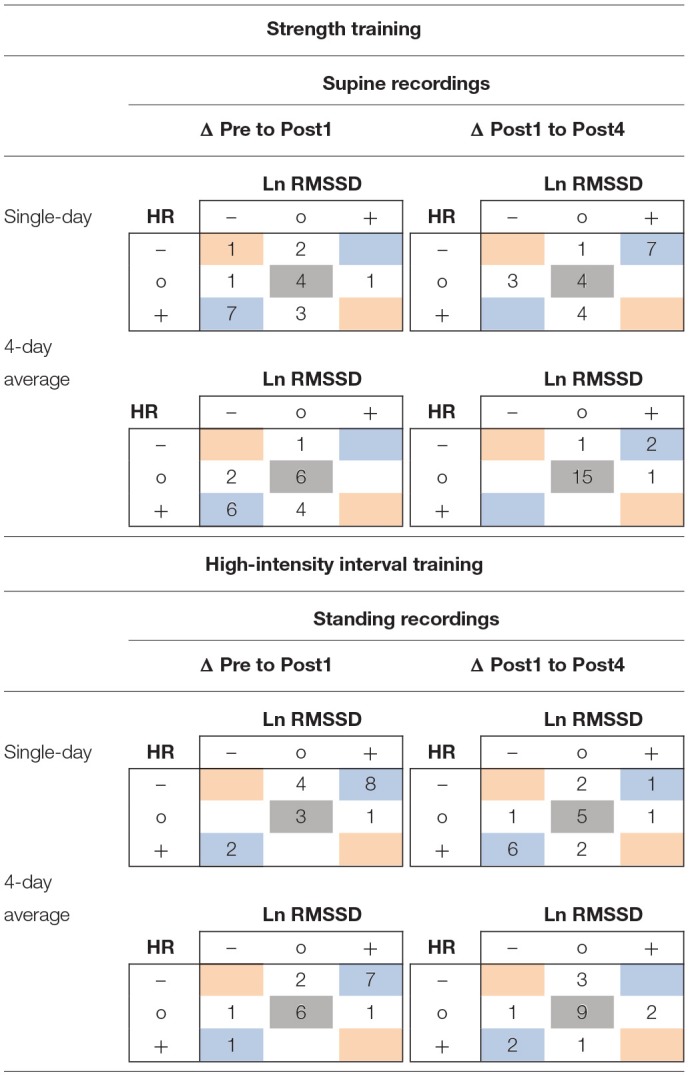
Example of 3 × 3 tables for individual response classification for changes from Pre to Post1 and Post1 to Post4 of resting heart rate and Ln RMSSD using single-day and 4-day average values.

*Data in the 3 × 3 tables represent the number of observed individual responses classified for 9 possible response types (3 response types for heart rate and Ln RMSSD, respectively). HR, heart rate; Ln RMSSD, natural logarithm of root mean square of successive differences between adjacent beat to beat intervals; –, observed reduction > typical error (TE); o, observed changes < TE; +, observed increase > TE. Individual TE was used for heart rate measures (individual SD during 4-day baseline period)*.

## Discussion

The purpose of this study was to characterize changes in HR and vagal HRV in response to ST and HIIT overload and short-term recovery. The main finding was that the HR(V) patterns identified during active orthostatic testing displayed altered supine HR(V) following ST while standing measures remained stable. Following HIIT, standing HR(V) measures were deflected while supine measures remained unchanged. Both continuous and categorical associations between changes in HR(V) indices and performance changes were weak or unclear. Further, our data suggested that the use of rolling averages may improve statistical sensitivity to group changes compared to the use of single-day HR(V). However, in the present study, average values appeared to be detrimental for assessing individual short-term responses when using the TE as a response threshold, as the magnitude of day-to-day changes was reduced. This may compromise sensitivity by decreasing signal-to-noise ratio, resulting in a decreased magnitude of change compared to the baseline variability.

### Evidence of Autonomic Modulation in Response to Short-Term ST and HIIT Overload

During ST overload, on average, supine HR increased, and supine Ln RMSSD decreased. The effects were reversed during the recovery period. Standing HR(V) recordings remained unchanged. These observations suggest small, reversible, decreased parasympathetic activity (increased HR and decreased Ln RMSSD) for the supine resting condition. However, autonomic responsiveness to orthostatic stress remained unaffected suggesting that ST overload did not impair parasympathetic withdrawal and/or sympathetic activation in response to standing up. As previously reported, overload induced substantial changes in perceived stress and recovery (Hitzschke et al., 2017), creatine kinase and c-reactive protein (Hecksteden et al., 2016), and jump performance (Raeder et al., 2016). The findings concerning the supine recordings align with the results of a recent review on the effects of resistance exercise and training on HRV (Kingsley and Figueroa, 2016). This suggests a prolonged decrease in parasympathetic modulation in young, healthy adults following acute whole-body resistance exercise. For example, vagal HRV (i.e., high frequency power) was reduced for at least 24 h after trained weightlifters performed whole-body resistance exercises for 2 h (Chen et al., 2011). In addition, the weightlifters had impaired weightlifting performance and increased creatine kinase concentrations. Unfortunately, previous studies on HRV responses to acute resistance exercise in strength-trained subjects have either used substantially less session volume (González-Badillo et al., 2016; Pareja-Blanco et al., 2017) or reported only acute effects within a few hours after exercising (Kingsley et al., 2014; Figueiredo et al., 2015a; Figueiredo et al., 2015b).

In response to the HIIT microcycle, supine HR(V) recordings remained unchanged on average. However, standing HR decreased and standing Ln RMSSD increased, with reverse changes occurring during recovery. Similar to ST, HIIT overload induced substantial changes in perceived stress and recovery (Hitzschke et al., 2017), creatine kinase (Hecksteden et al., 2016), and jump performance (Wiewelhove et al., 2015), as previously reported. In combination with stable HR(V) in a supine position, the changes associated with a standing position reflect an attenuated dynamic cardiovascular response to quickly changing from a supine to standing position. These findings may be attributed to the so-called saturation phenomenon that occurs in the supine position and reduced vagal withdrawal and/or reduced sympathetic activity during orthostatic stress. The saturation phenomenon indicates a loss of the relationship between HR and vagal HRV. It was suggested to be ascribed to saturation of acetylcholine receptors at the myocyte level, which may further suppress respiratory heart modulation and thus reduce vagal HRV measures at low HR (Buchheit, 2014). The saturation phenomenon is generally associated with low HR—it can occur at an HR of ~60 bpm or lower (Kiviniemi et al., 2004; Plews et al., 2012, 2013). Additionally, it may be partially related to aerobic capacity (Kiviniemi et al., 2004). This hypothesis (i.e., the presence of saturation in the supine position) is supported by the supine baseline HR of 57 bpm (i.e., 4-day average) for the group and the fact that 14 of 18 athletes showed supine baseline HR below 60 bpm. The evidence indicated that HRV saturation was likely in four athletes (athletes U, Z, AA, AF), as both supine HR and Ln RMSSD were decreased post-overload, but not in a standing position (Supplementary Table 6, Supplementary Figure 4). Although it has been suggested that parasympathetic reactivation is primarily intensity-dependent and it may take at least 48 h for vagal HRV to be fully restored after high-intensity aerobic exercise, higher aerobic fitness may accelerate post-exercise cardiac autonomic recovery (Stanley et al., 2013). For example, vagal HRV following a HIIT session (i.e., 6 × 3 min intervals above ventilatory threshold 2) returned to the pre-exercise level within a few hours in trained and highly trained subjects ($\dot{V}$O_2_max: 60 ± 5, and 72 ± 5 ml/min/kg, respectively) (Seiler et al., 2007). This suggests that vagal HRV indices might even overcompensate within the hours or days following very intense or prolonged exercise (Hottenrott and Hoos, 2017). This hypothesis is supported by previous studies, where increased Ln RMSSD were observed after 2 to 3 weeks of endurance-based overload training (Le Meur et al., 2013; Bellenger et al., 2016b), where average between-session recovery was likely smaller than 24 h. Another possible explanation for the reduced HR and increased Ln RMSSD in standing position following overload, could be altered sympathetic nervous system activity (e.g., due to reduced catecholamine excretion or desensitization of cardiac beta-adrenergic receptors) (Lehmann et al., 1998), which would become more evident after an increase in sympathetic activity in response to orthostatic stress.

The results agree with a meta-analysis (Bellenger et al., 2016a) on the effects of adaptation to endurance training on markers of autonomic HR regulation. Similar to the effects in the HIIT group, this meta-analysis reported a small increase in RMSSD following short training periods (i.e., 2 or 3 weeks), which led to decreased exercise performance. In addition, previous studies reported that standing HRV may be more sensitive to training-related changes compared to supine HRV as orthostatic stress may overcome possible saturation effects (Le Meur et al., 2013; Bellenger et al., 2016b).

To our knowledge, this is the first study to report HR(V) responses to ST and HIIT overload and subsequent short-term recovery measured with daily active orthostatic tests. We observed different HR(V) patterns during the orthostatic tests in response to the two different training modes, which might reflect activity- and/or fatigue-specific autonomic modulations (Schmitt et al., 2015a). Changing from a supine position to an active standing position causes a stress response due to the gravitational shift of blood from the central venous system to the lower extremities. This leads to severe vagal withdrawal as well as an increase in sympathetic-mediated vasomotor activity in order to preserve arterial blood pressure and avoid cerebral hypoperfusion (Buchheit et al., 2009a; Hottenrott and Hoos, 2017). Since supine and standing HR and HRV measures are influenced by the involvement of different cardiopulmonary receptors, we performed both supine and standing recordings. The postural HR(V) profiles were fully independent and non-exchangeable in elite endurance athletes (Schmitt et al., 2015a,b). Our observations provide some support for these arguments, as only combining supine and standing recordings enabled us to identify possible vagal saturation during HIIT overload and to describe different autonomic modulations depending on the training modes.

In summary, the observed differences in within-group HR(V) changes between study arms could be caused by various factors. On the one hand, differences in demands and in acute responses between training modalities may be responsible for different HR(V) patterns. In general, endurance-based (dynamic) exercise is suggested to mainly induce volume load on the cardiac cavities, whereas ST (i.e., static exercise) induces mainly pressure load (Aubert et al., 2003; Barbier et al., 2006). Further, HIIT is characterized by substantially greater amount of aerobic metabolism compared to ST (Barbier et al., 2006), and we assume higher total energy expenditure in response to HIIT. It was also suggested that HIIT exercise reduces arterial stiffness, while vigorous ST exercise increases arterial stiffness (Li et al., 2015; Way et al., 2019). HIIT may also induce acute plasma volume expansion (Green et al., 1984; Buchheit et al., 2009b). From a plausibility point of view, the described training responses could explain the observed within-group changes and between-group differences in orthostatic HR(V) regulation to some degree. However, we are not aware of studies providing direct evidence for such relationships, and overload training studies using active orthostatic tests in ST-trained subjects are entirely missing (see Kingsley and Figueroa, 2016; Bhati et al., 2018). On the other hand, the between-group differences could also be ascribed to differences in subject characteristics between study arms, such as higher average aerobic capacity in HIIT participants (Table 1) and possible additional training-specific functional (e.g., autonomic regulation) or structural adaptations (e.g., cardiac hypertrophy or changes in intrinsic HR) (Dickhuth et al., 1987; Achten and Jeukendrup, 2003; Billman et al., 2015a; Boyett et al., 2017; Flannery et al., 2017). In general, training-related HRV changes are frequently considered to be a result of altered autonomic HR regulation. However, in the absence of supportive physiological measurements, it remains speculative whether and to which degree the observed changes within our study were caused by different functional changes in response to varying exercise demands, or differences in training history-related adaptation and aerobic fitness between study groups.

### Lack of a Clear Association Between Changes in Performance and HR(V) After ST and HIIT Overload Microcycles

Overall, the continuous associations (i.e., correlations) between changes in HR, HRV and performance were mainly unclear (i.e., the confidence intervals display a substantial overlap with positive and negative effects) or inconsistent in direction from Pre to Post1 and Post1 to Post4. Categorical associations (i.e., 3 × 3 tables) also showed weak agreement between changes in HR(V) and criterion performance when using the TE as a threshold value. Therefore, short-term changes in HR(V) may be a poor surrogate marker for discipline-specific performance following strenuous ST or HIIT microcycles at the individual level. These findings seem plausible for several reasons. First, previously reported correlation analyses between criterion performance and other possible surrogate measures (i.e., perceived stress and recovery, blood-borne markers, muscle contractile properties, and non-fatiguing performance tests) also revealed unclear (i.e., not statistically significant) associations (Wiewelhove et al., 2015; Hecksteden et al., 2016; Raeder et al., 2016; Hitzschke et al., 2017), and these measures show a more direct theoretical relationship to discipline-specific performance compared to HR(V). Secondly, Plews et al. (2014) observed only moderate correlations between changes in Ln RMSSD and running performance in trained triathletes following a three-week overload period. Thus, we conclude that either the overload training stimulus was too low or short in duration or that the selected (criterion) performance tests were too noisy (i.e., they had a suboptimal signal-to-noise ratio) to reveal clear associations.

In summary, HR(V) measures may indeed reflect training- and recovery-induced autonomic modulations, which could affect athletes' performance. In addition, it has been proposed that cardiac vagal modulation may rather indicate an athlete's capacity to adapt to (aerobic) exercise stimuli and is therefore a prerequisite for performance-related adaptation (Hautala et al., 2009). However, an athlete's ANS status is only one factor contributing to the complex nature of fatigue and performance, and it is unlikely that a single marker can accurately display changes in such multidimensional constructs (Meeusen et al., 2013; Bourdon et al., 2017; Coutts et al., 2018; Kellmann et al., 2018; Schneider et al., 2018).

### Using Rolling HR(V) Averages May Attenuate Sensitivity to Individual Short-Term Changes in Autonomic Modulation

The use of average HR(V) improved the sensitivity to group changes compared to the use of single-day values, as indicated by the reduced width of confidence intervals for the *d*_pre_ and increased *d*_diff_ effect sizes (Figure 3, Tables 4, 5, Supplementary Figure 1, Supplementary Tables 1, 2). This supports previous proposals to use rolling averages (Le Meur et al., 2013; Plews et al., 2014). Visual inspection of the individual HR(V) response panels (Figure 4, Supplementary Figures 4–5) suggests lower percentage changes in average values for several athletes. In addition, the number of athletes showing both unchanged HR and Ln RMSSD for average values was increased (Table 7, Supplementary Table 6). The approach we used to classify the HR(V) response indicated inverse effects at the individual level compared to the group level. Although this finding relies solely on descriptive evaluation, it appears reasonable, as averaging daily values attenuates day-to-day change. However, this can cause important information to be lost, especially since cardiac autonomic recovery may occur within 24 h in trained athletes (Seiler et al., 2007; Stanley et al., 2013). On the other hand, however, averaging HR(V) may be necessary to reduce the measurement error for daily HR(V) changes. This controversial issue could be explored by future studies.

## Limitations and Strengths of the Study

Several factors limit the generalizability of results beyond the utilized study model. We primarily focused on determining the changes in HR(V) that occur following ST and HIIT overload in trained subjects. It remains unclear whether HR(V) also provides valuable information in more moderate, normal training environments or in highly trained athletes, as training and recovery responses may be diminished and accelerated, respectively, in these circumstances. In addition, adaptive responses to intensified training may be characterized by changes in the magnitude of day-to-day fluctuation in Ln RMSSD (Flatt and Howells, 2019), but this was not assessed in the current study. Further, as previously discussed in detail (Hecksteden et al., 2016), the comparability of HR(V) responses to ST vs. HIIT is constrained by the generic challenge of matching workload and fatigue levels between the different modes of exercise. Moreover, recruitment of performance-matched control groups for the (in total) three different study arms was not feasible due to limited time and resources. To verify our findings, randomized (crossover) trials with a priori optimized sample size are desired. Another limitation is that, despite an initial survey of medication and nutritional supplementation, it was neither possible for us to control the intake of HR(V) influencing medication throughout the study period, nor the consumption of caffeinated beverages immediately prior to orthostatic testing. Finally, as HR(V) is an indirect measure of cardiac autonomic modulation, interpretations regarding underlying physiological mechanisms should be treated with caution.

We tried to overcome several limitations by assessing the fatigue responses using criterion performance tests and utilizing repeated testing [i.e., daily HR(V) recordings] with a single-subject A-B-A withdrawal design (Kinugasa et al., 2004; Barker et al., 2011). The latter enabled us to describe HR(V) changes more precisely by considering individual day-to-day variation, which is not possible in a simple pre-post design. Furthermore, week-to-week reliability was determined beforehand in our laboratory (Wiewelhove et al., 2015; Raeder et al., 2016). It indicates random variation in criterion performance and may partially be a substitute for the presence of control groups (Hopkins, 2015a; Hecksteden et al., 2018). To our knowledge, comparative assessment of daily resting HR(V) for ST and HIIT during active orthostatic tests in a methodologically consistent design is a unique approach that offers novel insights into HR(V) responses.

## Conclusion

Daily HR(V) monitoring with active orthostatic tests displayed altered supine HR(V) measures for ST- and altered standing HR(V) measures for HIIT short-term overload. HR(V) measures remained unchanged in the respective other recording position. These autonomic patterns may not be discovered by supine or standing measures alone. However, HR(V) changes were not consistently related to short-term performance changes, which limits their usefulness as surrogate measures for ST or HIIT performance in overload microcycles. Moreover, the use of rolling averages may attenuate the sensitivity to individual short-term autonomic modulations, despite improving sensitivity at the group level. To provide further guidance for sports practice, future studies should utilize repeated intervention designs (Hecksteden et al., 2015) with appropriate baseline recordings to determine the consistency of acute and short-term HR(V) responses, as previously done for long-term adaptation to training (Plews et al., 2013).

## Practical Applications

The combined assessment of HR(V) measures in supine rest and following quickly standing up using orthostatic tests can provide unique and more comprehensive insights into athletes' autonomic HR regulation compared to either supine or standing recordings in isolation. Although divergent autonomic patterns might be observed following various training demands, it should be acknowledged that HR(V) measures may not mimic individual short-term performance changes. Furthermore, based on our results, we encourage practitioners to (also) analyze single-day HR(V) measures when assessing short-term training responses. Finally, day-to-day variability and training response varies substantially between athletes, which further complicates HR(V)-guided training prescription. In summary, we believe that it is still advisable first to gain experience at an individual level through pure observation in order to avoid inappropriate training adjustments due to overly simplistic training-response models.

## Ethics Statement

The investigation was approved by the ethics committee of the medical faculty of the Ruhr University Bochum and was conducted according to the guidelines of the Declaration of Helsinki. All subjects participated in the study voluntarily, were free to withdraw without penalty at any time, and provided written informed consent.

## Author Contributions

CS prepared the original manuscript, figures and tables and analyzed the data. CS, AAF, and OH interpreted the results. TW, CR, AAF, OH, LH, and AF assisted with writing and editing the manuscript, figures and tables. TW and CR performed the experiment. OS calculated the heart rate variability indices from raw data files. TW, CR, MK, TM, MP, and AF conceived and designed the experiment.

### Conflict of Interest Statement

The authors declare that the research was conducted in the absence of any commercial or financial relationships that could be construed as a potential conflict of interest.

## Supplementary Material

Table 1.xlsx Supplementary Tables.

Data Sheet 1.xlsx Data Sheet.

Data Sheet 2.zip Data analysis and statistical analysis.

Data Sheet 3.docx Instructions heart rate recordings.

Image 1.pdf Supplementary Figures.

## Abbreviations

1RM: one-repetition maximum
ANS: Autonomic nervous system
CL: Confidence limits
ddiff: standardized mean difference using SD of differences
dpre: standardized mean difference using between-subject Pre-test SD
HIIT: High-intensity interval training
HR: Heart rate
HRV: Heart rate variability
HR(V): Heart rate and heart rate variability
Ln RMSSD: Natural logarithm of the RMSSD
Ln RMSSD/RR: Ln RMSSD to R-R interval ratio
MVIC: Maximum voluntary isometric contraction
Pre: Baseline testing 1 day prior training microcycle
Post1: Follow-up testing 1 day post overload
Post4: Follow-up testing 4 days post overload
RMSSD: square root of the mean squared differences of successive normal R-R intervals
RSA: Repeated sprint ability
SD: Standard deviation
ST: Strength training
SWC: Smallest worthwhile change
TE: Typical error
V_IFT_: Peak velocity during the 30-15 Intermittent Fitness Test.
