# Supplementary material for: Heart Rate Variability Monitoring During Strength and High-Intensity Interval Training Overload Microcycles
Source: Front Physiol. 2019 May 22;10:582. doi: 10.3389/fphys.2019.00582 (PMC6538885; doi:10.3389/fphys.2019.00582)
Supplement: Data Sheet 3.docx — Instructions heart rate recordings. [file Data_Sheet_3.docx]

Supplementary Material – Instructions heart rate recordings

# Heart rate recording during training – Polar RS800CX^[[1]](#footnote-1)^

**Initial position:** Time and date are displayed.

(if not, then press the "Stop" button on the bottom left until the time and date appear).

**Training preparation:** Moisten the belt slightly. Press "OK" on the big red button in the middle (to show current heart rate).

**Start of recording:** When heart rate is displayed, press the red button once again (stopwatch starts) when you start exercising.

**Stop and save session recording:**

Press "Stop button" on the bottom left until the time and date appear.

**Important:** Without consultation, no changes must be made to the settings of the watch!

# Heart rate variability recording

In the morning immediately after waking up heart rate variability (HRV) is measured using the Polar RS800CX. The recording takes approximately 15 minutes, of which you lie in bed for 7 minutes and stand 5 minutes without moving. Between waking up and recording, it is a mandatory to use the bathroom. If you do not need to use the bathroom, please simulate it.

Put on the watch (RS800CX) and the chest strap (WearLink) in the bathroom.

Lie back in bed after putting on the watch and the chest strap. After finding a comfortable supine position, start the heart rate recording, **as if you are recording a training session.**

Both when measuring while lying down and standing, try to move as little as possible. Keep your eyes open and try to breathe calmly. After 7 minutes, stop recording and get out of bed. Now start the recording a second time while standing. Remain in an upright position. The arms hang sideways next to the body, the knees are slightly bent, and the view is directed forward. After another 5 minutes stop the recording.

**The recording works in the same way as recording a training session. It is important that the red OK-button is pressed twice until the stopwatch is running. Otherwise, no recording will take place.**

1. No training recordings were analyzed within the strength training and high-intensity interval training study arms [↑](#footnote-ref-1)
