# Supplementary material for: Heart Rate Variability Monitoring During Strength and High-Intensity Interval Training Overload Microcycles
Source: Front Physiol. 2019 May 22;10:582. doi: 10.3389/fphys.2019.00582 (PMC6538885; doi:10.3389/fphys.2019.00582)
Supplement: Image 1.pdf — Supplementary Figures. [file Image_1.pdf]

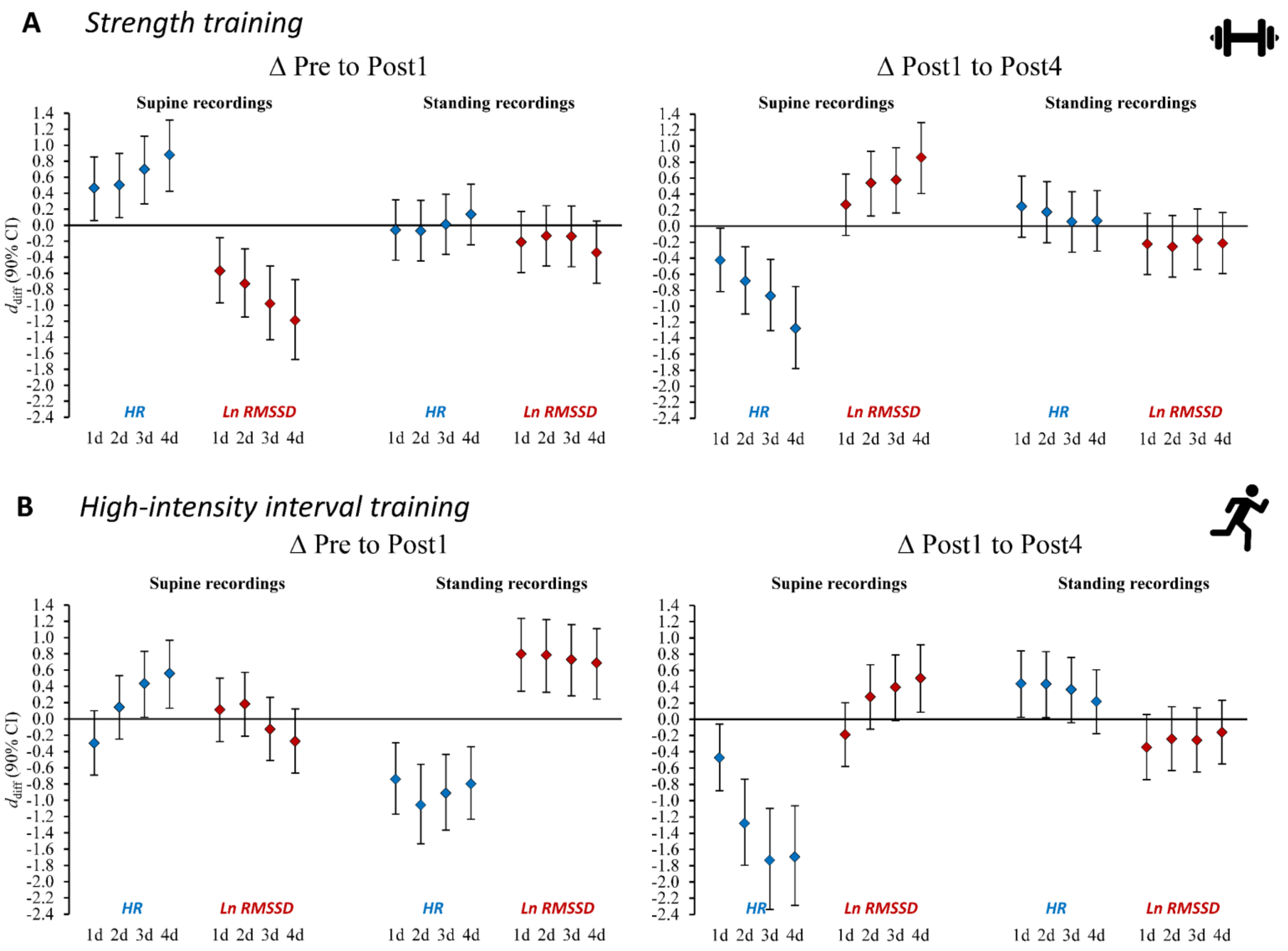

Supplementary Figure 1 | Standardized mean differences (d_{diff}) for changes in heart rate (variability) measures from Pre to Post1 and from Post1 to Post4. HR(V) measures are provided as single-day values and 2–4-day rolling averages. HR: heart rate, Ln RMSSD: natural logarithm of root mean square of successive differences between adjacent beat to beat intervals.

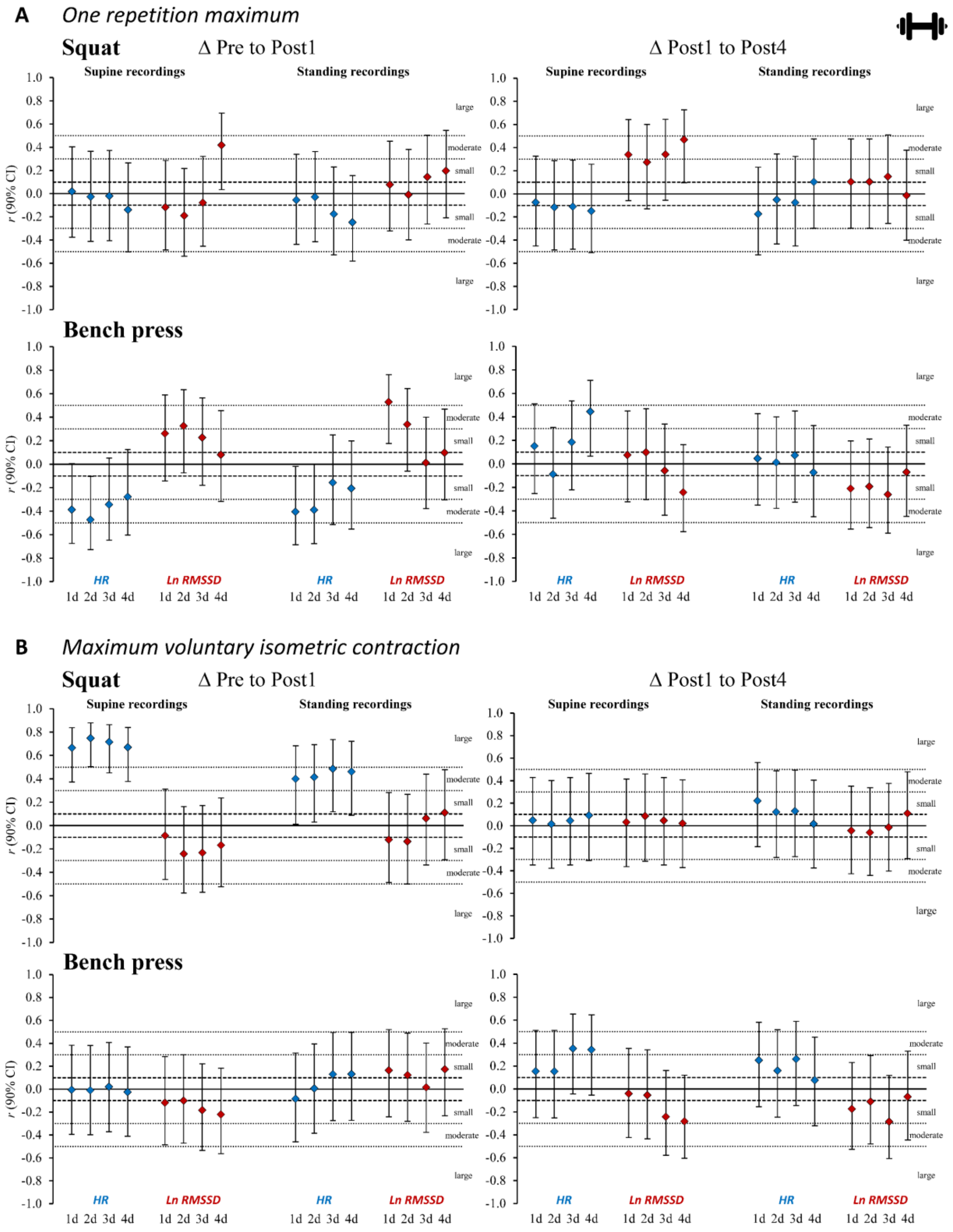

Supplementary Figure 2 | Correlations between performance and heart rate (variability) measures for percentage changes from Pre to Post1 and from Post 1 to Post4 for the strength training group. HR(V) measures are provided as single-day values and 2–4-day rolling averages. HR: heart rate, Ln RMSSD: natural logarithm of root mean square of successive differences between adjacent beat to beat intervals.

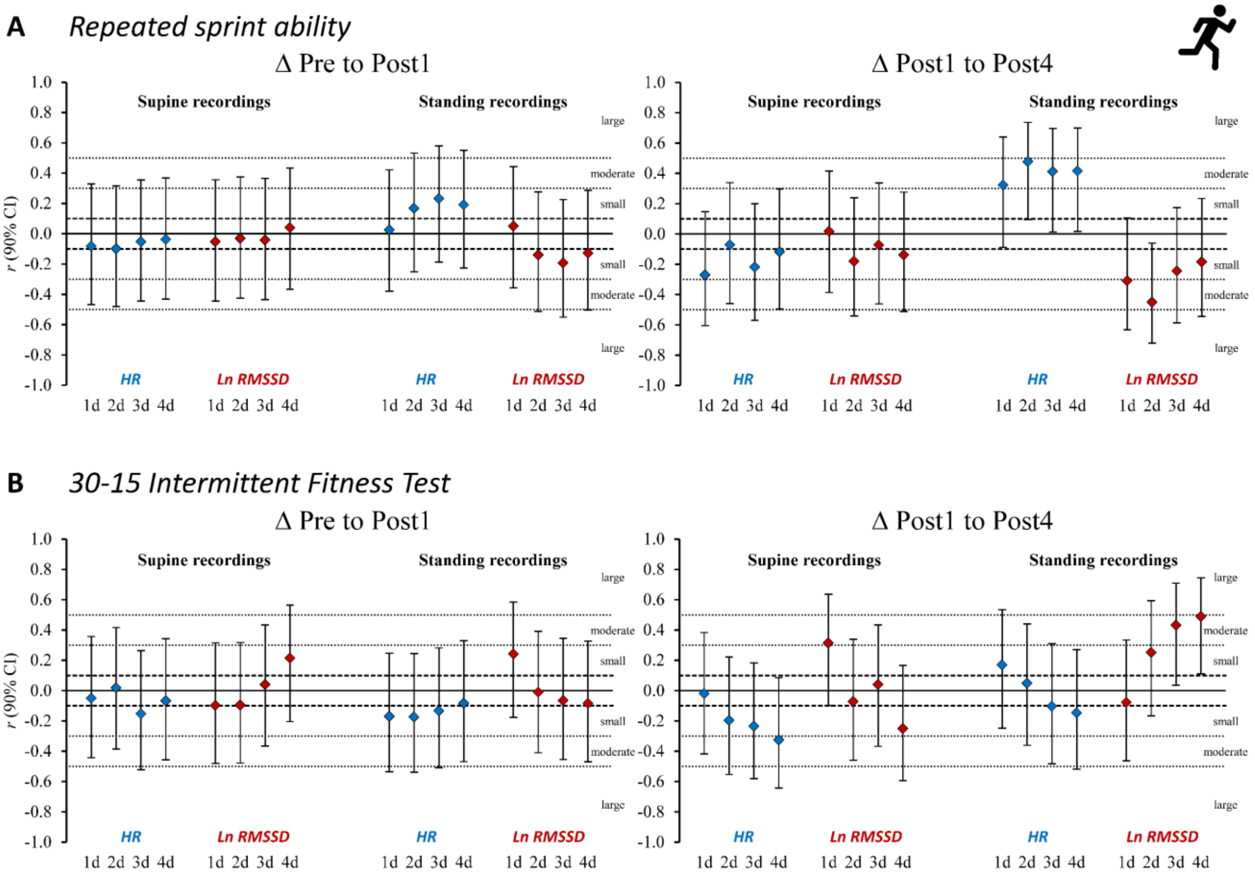

Supplementary Figure 3 | Correlations between performance and heart rate (variability) measures for percentage changes from Pre to Post1 and from Post 1 to Post4 for the high-intensity interval training group. HR(V) measures are provided as single-day values and 2–4-day rolling averages. HR: heart rate, Ln RMSSD: natural logarithm of root mean square of successive differences between adjacent beat to beat intervals.

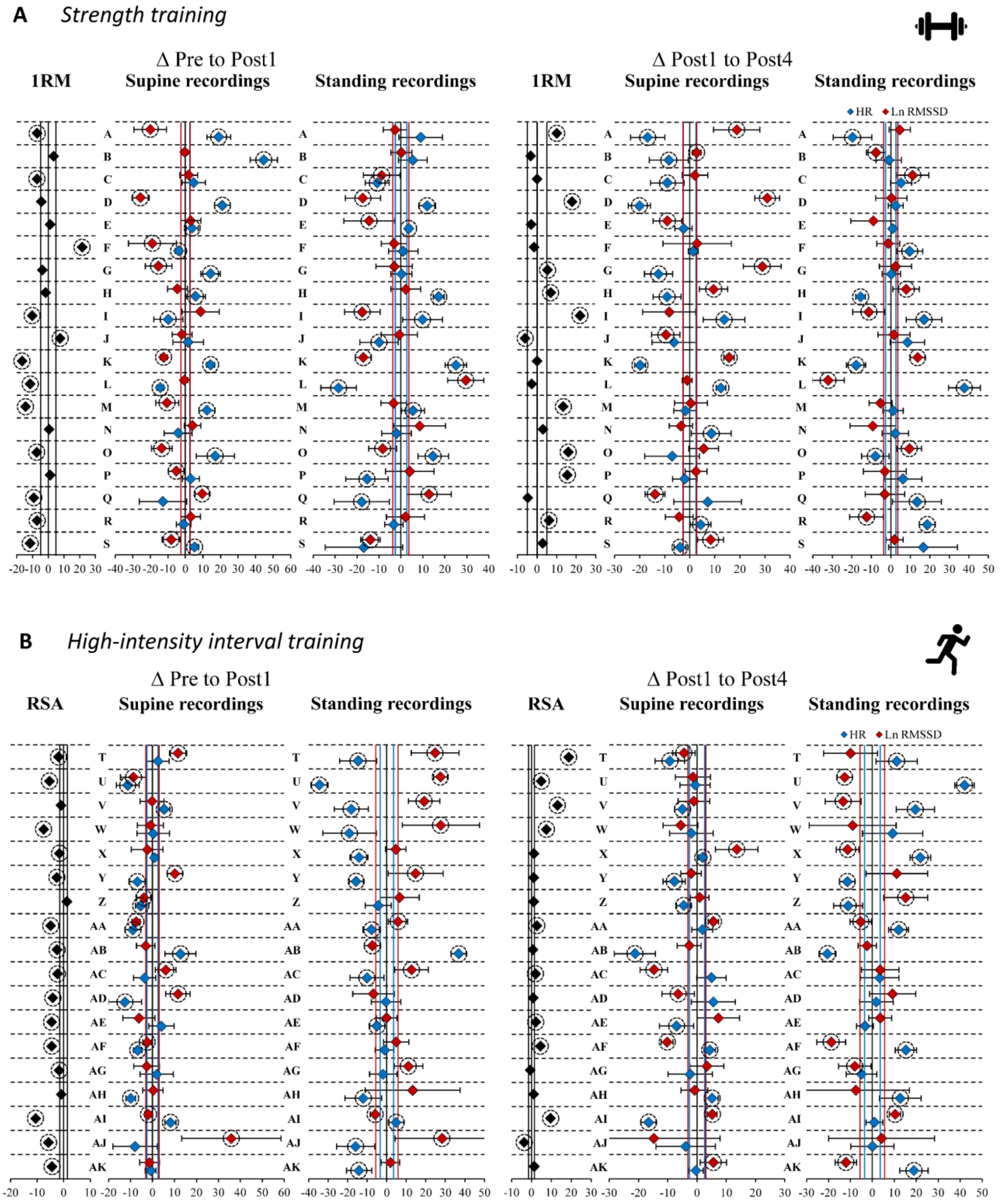

Supplementary Figure 4 | Individual responses as percentage changes in criterion performance and single-day HR(V) measures. 1RM: one repetition maximum, RSA: repeated sprint ability, HR: heart rate, Ln RMSSD: natural logarithm of root mean square of successive differences between adjacent beat to beat intervals. Vertical lines: group-based typical error (TE) for RSA (black), smallest worthwhile change (see Table 3) in HR (blue), and Ln RMSSD (red). Error bars: individual TE (4-day baseline SD). Dashed circles: changes exceed the ±TE.

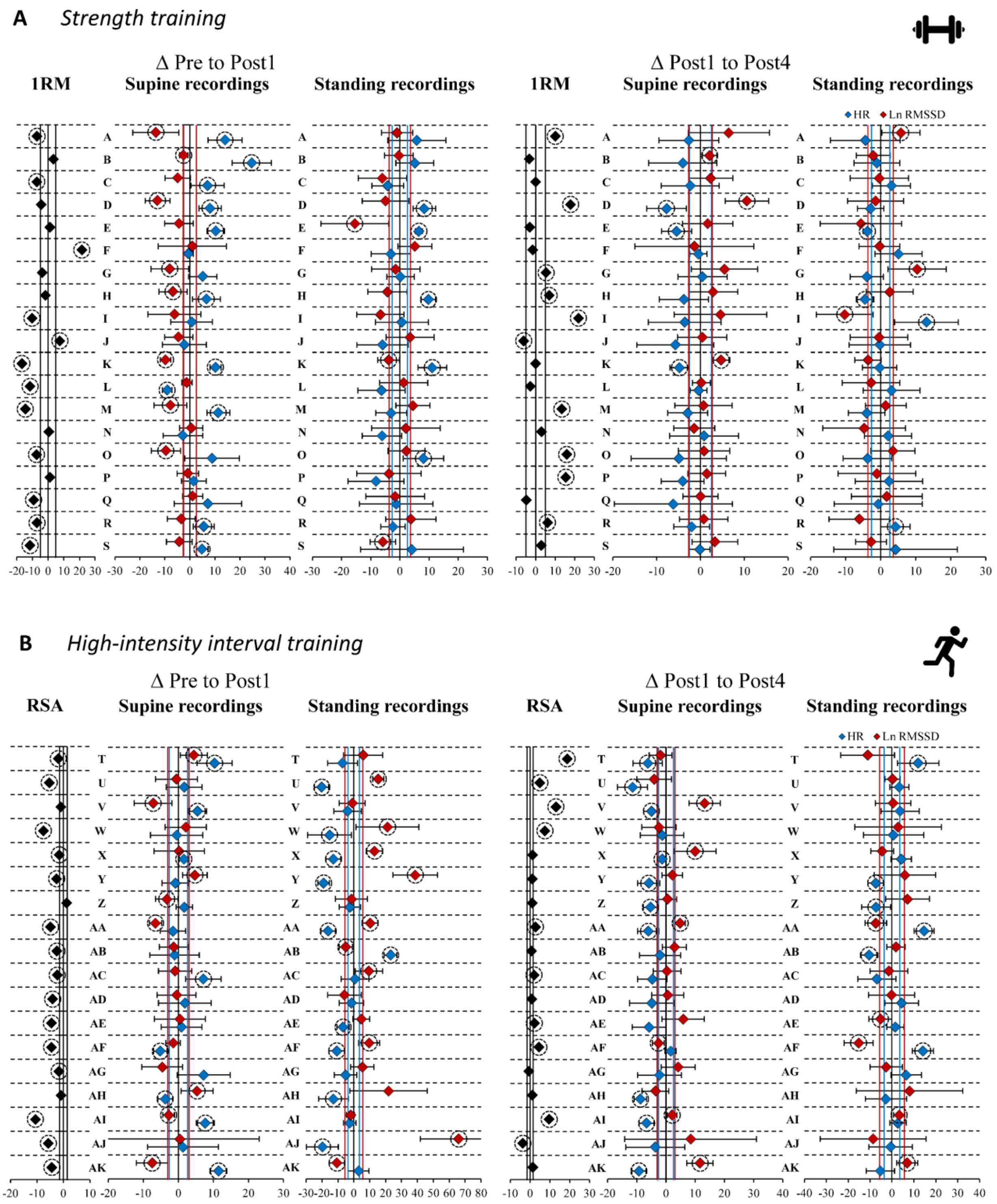

Supplementary Figure 5 | Individual responses as percentage changes in criterion performance and 4-day averaged HR(V) measures. 1RM: one repetition maximum, RSA: repeated sprint ability, HR: heart rate, Ln RMSSD: natural logarithm of root mean square of successive differences between adjacent beat to beat intervals. Vertical lines: group-based typical error (TE) for RSA (black), smallest worthwhile change (see Table 3) in HR (blue), and Ln RMSSD (red). Error bars: individual TE (4-day baseline SD). Dashed circles: changes exceed the ±TE.
